# Comparative proteomics analysis of differential proteins in respond to doxorubicin resistance in myelogenous leukemia cell lines

**DOI:** 10.1186/s12953-014-0057-y

**Published:** 2015-01-22

**Authors:** Shi Qinghong, Gao Shen, Song Lina, Zhao Yueming, Li Xiaoou, Wu Jianlin, He Chengyan, Li Hongjun, Zhao Haifeng

**Affiliations:** Jilin University China-Japan Union Hospital, Changchun, 130033 China; Jiamusi University, Jiamusi, 154002 China; Tumor Hospital of Jilin Province, Changchun, 130021 China; State Key Laboratory for Quality Research in Chinese Medicines, Macau University of Science and Technology, Avenida Wai Long, Taipa, Macau, China

## Abstract

**Background:**

Chemoresistance remains a significant challenge in chronic myelogenous leukemia (CML) management, which is one of the most critical prognostic factors. Elucidation the molecular mechanisms underlying the resistance to chemoresistance may lead to better clinical outcomes.

**Results:**

In order to identify potential protein targets involved in the drug-resistant phenotype of leukemia, especially the chronic myelogenous leukemia (CML), we used a high-resolution “ultra-zoom” 2DE-based proteomics approach to characterize global protein expression patterns in doxorubicin-resistant myelogenous leukemia cells compared with parental control cells. Ultra-high resolution of 2DE was achieved by using a series of slightly overlapping narrow-range IPG strips during isoelectric focusing (IEF) separation. A total number of 44 proteins with altered abundances were detected and identified by MALDI-TOF or LC-MS/MS. Among these proteins, enolase, aldolase, HSP70 and sorcin were up-regulated in doxorubicin-resistant myelogenous leukemia cell line, whereas HSP27 was down-regulated. Some of the results have been validated by Western blotting. Both enolase and aldolase were first reported to be involved in chemoresistance, suggesting that process of glycolysis in doxorubicin-resistant myelogenous leukemia cells was accelerated to some extent to provide more energy to survive chemical stress. Possible roles of most of the identified proteins in development of chemoresistance in myelogenous leukemia cells were fully discussed. The results presented here could provide clues to further study for elucidating the mechanisms underlying drug resistance in leukemia.

**Conclusions:**

As a whole, under the chemical stress, the doxorubicin-resistant myelogenous leukemia cells may employ various protective strategies to survive. These include: (i) pumping the cytotoxic drug out of the cells by P-glycoprotein, (ii) increased storage of fermentable fuel, (iii) sophisticated cellular protection by molecular chaperones, (iv) improved handling of intracellular calcium, (v) increased glucose utilization via increased rates of glycolysis. In the present study, proteomic analysis of leukemia cells and their drug resistant variants revealed multiple alterations in protein expression. Our results indicate that the development of drug resistance in doxorubicin-resistant myelogenous leukemia cells is a complex phenomenon undergoing several mechanisms.

**Electronic supplementary material:**

The online version of this article (doi:10.1186/s12953-014-0057-y) contains supplementary material, which is available to authorized users.

## Background

Doxorubicin, also known as hydroxydaunorubicin, is a drug commonly used in the treatment of a variety of cancers, especially acute myelogenous leukemia (AML) and chronic myelogenous leukemia (CML) [1]. However, resistance to doxorubicin is often observed in patients with leukemia, resulting in failure in chemotherapy. Such chemoresistance is a phenomenon found in many types of cancers including hematological malignancies (leukaemia and lymphoma), many kinds of carcinoma (solid tumors) and soft tissue sarcomas. Once the cancers develop chemoresistance to a distinct drug, they often display low sensitivity to a variety of other chemotherapy drugs and might not respond to these drug therapies, especially in the case of multi-drug resistance in chronic myelogenous leukemia (CML).

Efforts have been made to reveal the molecular mechanisms underlying the development of chemoresistance, particularly multi-drug resistance (MDR) in CML. Early studies had established that drug resistant CML is mediated by P-glycoprotein (Pgp), a protein that functions as a drug efflux pump [2]. Glutathione S transferase (GST) is another important protein found to be associated with MDR [3,4], of which the expression is often observed to be up-regulated in drug resistant cell lines. A recent study indicated that CXCL12 could enhance chemoresistance of K562 cells to doxorubicin by increasing the expression of CXCR4, a seven-transmembrane G-protein-coupled chemokine receptor [5]. Apart from above-mentioned proteins, several other proteins such as sorcin [6,7], survivin [8] and endothelin-1 [9], etc., have been observed to be associated with the development of chemoresistance in CML. Accumulated evidence has shown that these proteins are involved in multiple different pathways and often interact with each other, indicating that the mechanisms mediating drug resistance in CML are multifaceted and still not clearly defined.

Due to the complexity of the changes occurring upon the development of drug resistance, it is of great importance to apply more comprehensive approaches to decipher the codes embedded in drug resistance in leukemia. During the past decade, proteomics has become the powerful tool to perform large scale analysis of complex protein mixtures [10-12]. Two-dimensional gel electrophoresis (2-DE) combined with mass spectrometry (MS) has been one of the most widely used tools in proteomics studies.

Specifically, 2-DE separates proteins according to their isoelectric point (pI) and molecular mass (Mr), both of which are orthogonal parameters of a protein molecule. For separation of a given proteome at protein level, 2-DE has always been the most powerful method to study protein expression and function in living organisms and diseases [13,14]. In a typical 2-DE experiment, immobilized pH gradients (IPGs) with wide pH range (e.g., pH 3–10 or pH 3–11) are used to resolve complex protein mixtures as the first dimensional separation, usually allowing over 1000 protein spots to be visualized on a standard 2-D gel. To achieve better separation in the first dimension of 2-DE, a series of IPG stripes with overlapping narrow pH range such as ultra-zoom IPG stripes (e.g., pH 4.5-5.5 and pH 5.5-6.7) may be used, allowing much more protein spots to be detected with increased resolution [15,16].

In the present study, in order to effectively search for more proteins involved in the development of chemoresistance in CML, we utilized ultra-zoom gels to analyze differences in global protein expression in doxorubicin-resistant myelogenous leukemia cell line K562/A02 and its parental control cell line K562. A series of IPGs with pH range 3.9-5.1, 4.7-5.9, 5.5-6.7 and 6.3-8.3 were used to obtain high resolution 2-DE gels. Up to 44 differentially expressed proteins were identified. The involvement of the identified proteins in the development of drug resistance in leukemia cells was discussed.

## Results

### Cytotoxicity assay

**Figure 1:**
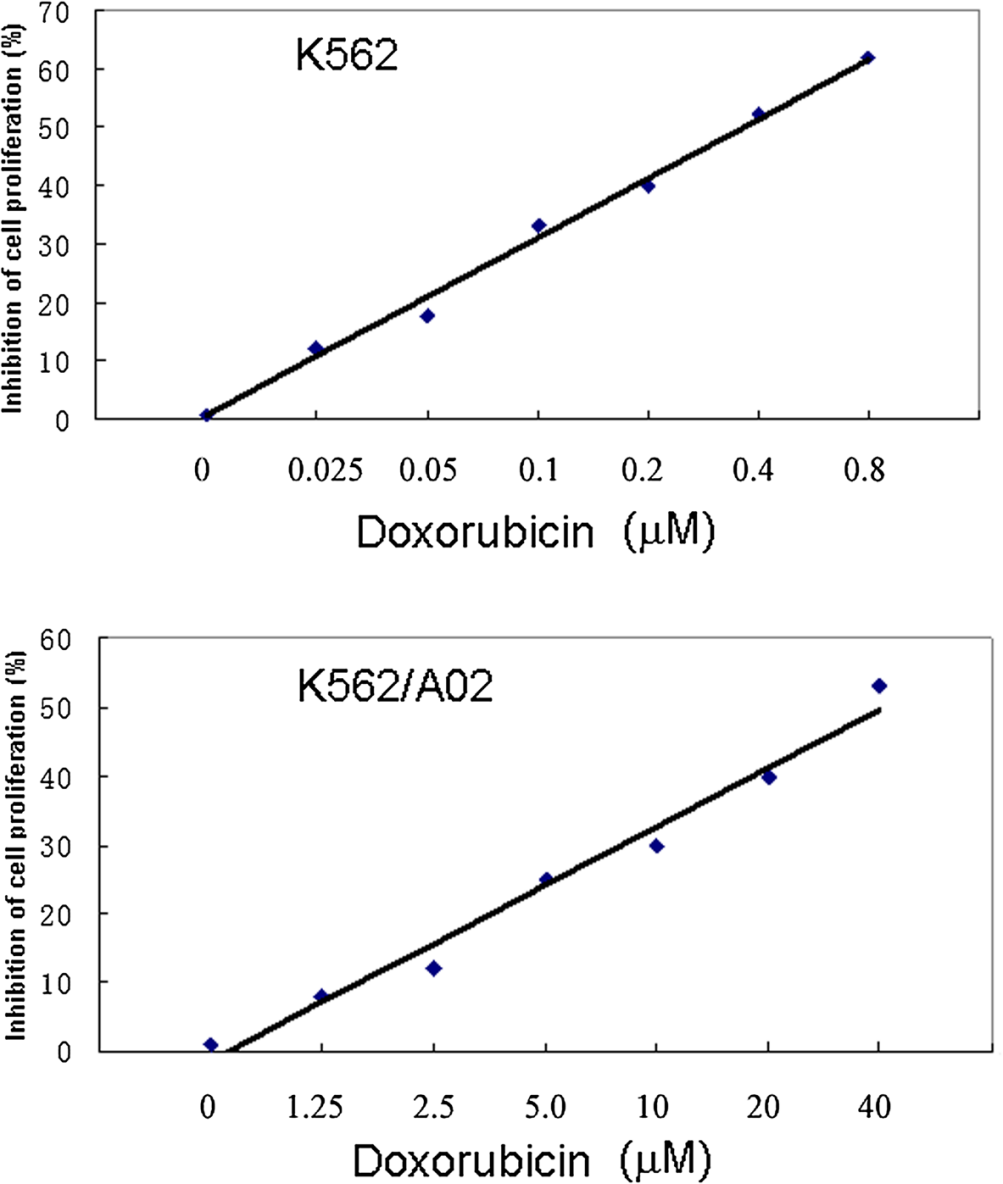
**Cell proliferation assays for both K562/A02 and K562 cultured in the presence or absence of doxorubicin.**

Cell proliferation assays for both K562/A02 and K562 cultured in the presence or absence of doxorubicin were performed to investigate the drug-resistance characteristics of K562/A02 cells, as well as the viability of K562 cells under chemical stress (Figure 1). Then IC50 values of doxorubicin for both K562/A02 and K562 were calculated. As expected, the IC50 of K562/A02 cells was much greater than that of K562 cells.

### 2-DE-based proteomics analysis using ultra-zoom IPG strips

The protein expression pattern of the drug resistant K562/A02 cell line was compared to that of the parental K562 cell line using PDQuest software (Version 7.1.1; Bio-Rad). For each cell line, quadruplet of gel images with identical pI range in the IEF separation was assigned for a group using PDQuest software. Thus, two groups, namely one containing the gels of the chemoresistant cell line K562/A02 and one containing the gels of the parental sensitive cell line K562 were summarized to a statistical analysis set. The average quantity of each quadruplet of matched spots in the replicate gels in each group was calculated and the differentially expressed protein spots with p value smaller than 0.05 through Student’s t-test were registered. From the statistically differentially expressed protein spots, only those protein spots with quantitative difference between the two groups greater than 2-fold in magnitude were subject to further identification by MS and MS/MS analyses.

**Figure 2:**
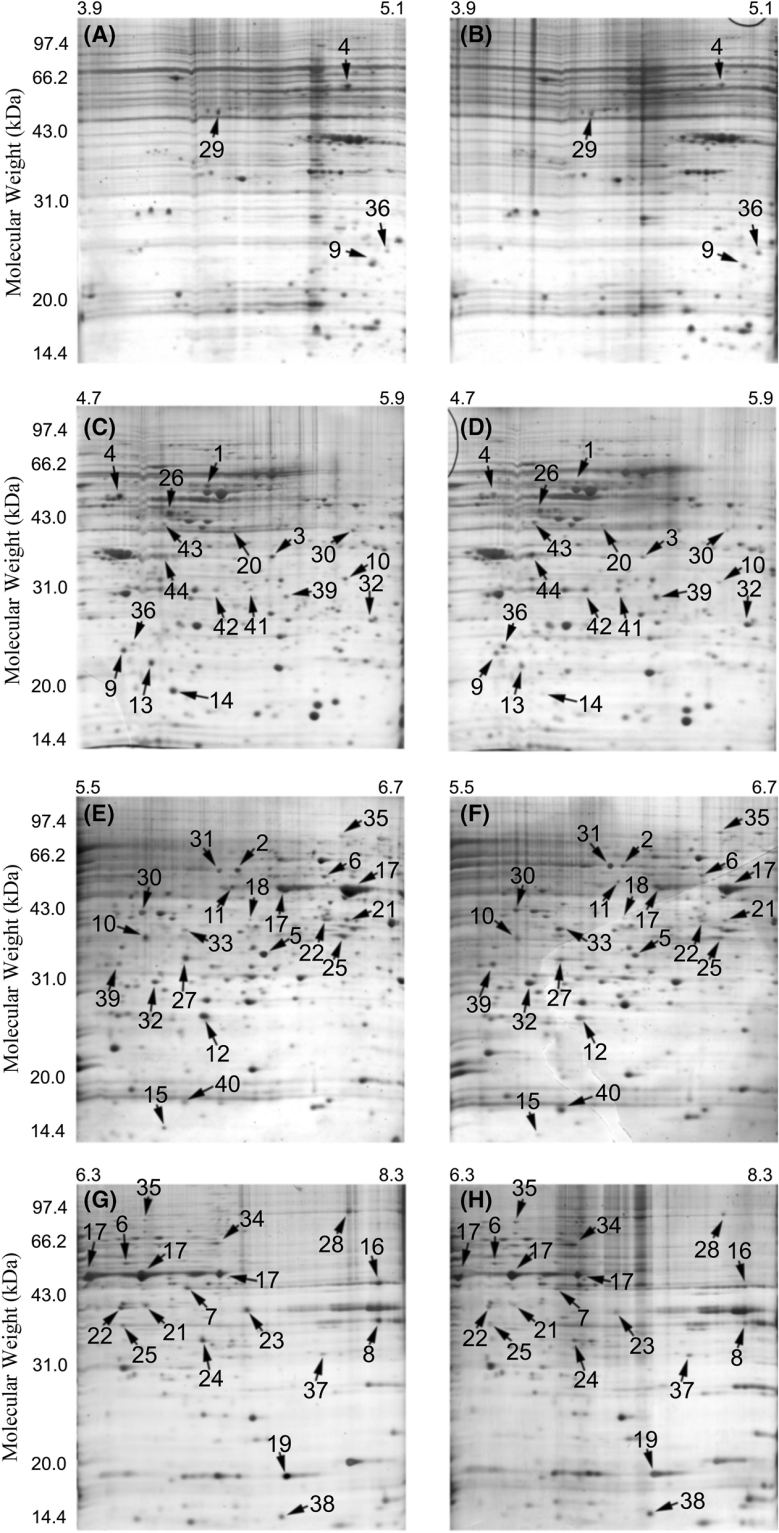
**A group of pairs of protein 2-D maps of doxorubicin-resistant myelogenous leukemia cell line K562/A02 (left) and its parental control cell line K562 (right) with sample loading of 0.6 mg protein each.** The isoelectric focusing was carried on 17 cm IPG strips with a pH range of either 3.9-5.1 **(A, B)**, 4.7-5.9 **(C, D)**, 5.5-6.7 **(E, F)** or 6.3-8.3 **(G, H)**. For the second dimension, acrylamide gels of 12.5% were used. The gels were stained by Colloidal Coomassie blue G-250 over night. Numbers associated with the spots on the gel images refer to the identified proteins listed in Table 1.

**Figure 3:**
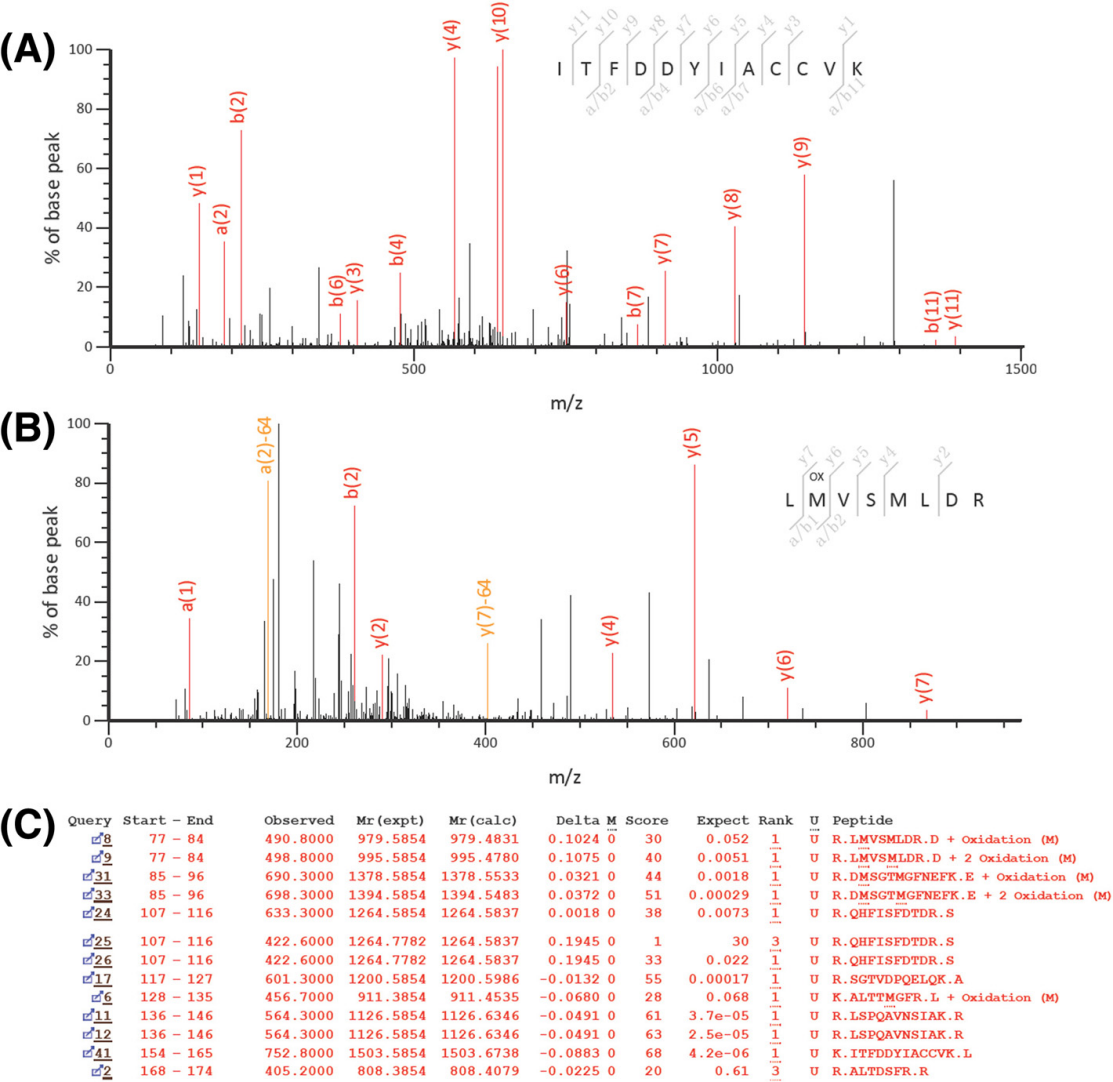
**Identification of sorcin by mass spectrometry analysis and database searching. (A)** MS/MS spectrum of a doubly-charged peak at m/z 752.8. The corresponding peptide is identified as ITFDDYIACCVK (154–165). **(B)** MS/MS spectrum of a doubly-charged peak at m/z 490.8. The corresponding peptide is identified as LMVSMLDR (77–84), in which the methionine is oxidized. **(C)** Identification of sorcin by MS/MS analysis. The data were searched against SwissProt database through Mascot engine.

Figure 2 shows a group of gel images of the drug resistant cell line K562/A02 (Figure A, C, E and G) and the parental cell line K562 (Figure B, D, F and H), in which a series of ultra-zoom IPG strips with slightly overlapping pH range (pH 3.9-5.1, 4.7-5.9, 5.5-6.7 and 6.3-8.3, 17 cm, Bio-Rad) were used in the first dimensional IEF procedure to greatly increase resolution of IEF. The analysis revealed 44 protein changes between the drug resistant cells and their parental cells, as listed in Table 1. Among the 44 proteins, 30 were up-regulated in drug resistant cells and 14 were down-regulated. Because the IPG strips were slightly overlapped with their neighboring IPG strips, several proteins were detected on more than one gel such as protein 4, 9, 36, 10, 32, 6, 17, 21, 22, etc., of which the robustness of our proteomics analysis was significantly enhanced. For example, detection of protein 9 on gels with IPG strip (pH 3.9-5.1) was confirmed by detection of the same protein on gels with IPG strip (pH 4.7-5.9). All the protein spots were identified by either PMF or MS/MS method alone or the both. Notably, three protein spots (protein 17) were identified as enolase 1, which were believed to be three isoforms of the same protein. From the comparative proteomics analysis of the doxorubicin-resistant myelogenous leukemia cells and their parental cells, protein 14 on Figure 1 was observed to be highly differentially expressed, which was identified as sorcin, a soluble resistance-related calcium-binding protein. The detailed information regarding to the identification of sorcin by mass spectrometry, as well as the corresponding MS/MS spectra, was shown in Figure 3. Protein spot 43, identified as ATP synthase subunit beta, was found to be down-regulated in K562/A02, of which the result was well consistent with that of a previous study using K562/A02 [17]. The original MS/MS data have been provided in “MS/MS data” file as Additional file 1.

**Table 1:** **Summary of differentially expressed proteins**

**Spot no.**	**Accession number**	**Protein name**	**Function**	**Identification method**	**Unique peptides by MS/MS**	**Theoretical pI/Mw (kDa)**	**Mascot score**	**Diffenrential ratio**
Proteins with increasing spot intensity								
1	P08107	Heat shock 70 kDa protein 1A/1B	Unfolded protein binding	PMF, MS/MS	8	5.47/70.05	98	2.03 (2.55)*
2	P17987	T-complex protein 1 subunit alpha	Molecular chaperonin	MS/MS	17	5.80/60.34	103	3.91
3	P50453	Serpin B9	Cysteine-type endopeptidase inhibitor activity involved in apoptotic process	PMF, MS/MS	4	5.61/42.40	67	2.34
4	P08670	Vimentin	Double-stranded RNA binding	PMF		5.05/53.65	122	4.84
5	P04083	Annexin A1	Calcium ion binding	PMF		5.85/38.71	132	2.94
6	P20839	Inosine-5′-monophosphate dehydrogenase 1	IMP dehydrogenase activity, metal ion binding	PMF, MS/MS	5	6.43/55.41	116	2.17
7	P12532	Creatine kinase U-type	ATP binding	PMF, MS/MS	3	7.31/43.08	89	3.15
8	P04075	Fructose-bisphosphate aldolase A	Fructose-bisphosphate aldolase activity	PMF		8.30/39.42	142	4.14
9	P52565	Rho GDP-dissociation inhibitor 1	GTPase activator activity	PMF, MS/MS	5	5.01/23.21	127	3.06
10	P07195	L-lactate dehydrogenase B chain	L-lactate dehydrogenase activity	PMF		5.71/36.64	84	2.58
11	V9HW96	Chaperonin containing TCP1, subunit 2 (Beta), isoform CRA_b	Molecular chaperonin	PMF		6.01/57.49	133	2.09
12	P30041	Peroxiredoxin-6	Antioxidant activity	PMF, MS/MS	8	6.00/25.03	93	3.56
13	O00299	Chloride intracellular channel protein 1	Chloride channel activity	PMF, MS/MS	9	5.09/26.92	122	4.19
14	P30626	Sorcin	Calcium channel regulator activity	PMF, MS/MS	4	5.32/21.68	142	50.70
15	P31949	Protein S100-A11	Calcium ion binding	PMF, MS/MS	3	6.65/11.74	89	2.92
16	P00558	Phosphoglycerate kinase 1	Phosphoglycerate kinase activity	PMF		8.30/44.61	91	3.13
17	P06733	Alpha-enolase	Phosphopyruvate hydratase activity	PMF, MS/MS	22	7.01/47.17	123	2.17 (2.93)*
18	Q9NR45	Sialic acid synthase	N-acetylneuraminate synthase activity	PMF		6.29/40.31	97	2.04
19	P62837	Ubiquitin-conjugating enzyme E2 D2	Ubiquitin-protein ligase activity	PMF, MS/MS	4	7.69/16.74	137	2.46
20	P05787	Keratin, type II cytoskeletal 8	Scaffold protein binding	PMF, MS/MS	9	5.52/53.71	152	2.03
21	Q14320	Protein FAM50A	Poly(A) RNA binding	PMF, MS/MS	6	6.39/40.24	104	2.38
22	P09972	Fructose-bisphosphate aldolase C	Fructose-bisphosphate aldolase activity	PMF		6.41/39.46	79	2.11
23	Q08752	Peptidyl-prolyl cis-trans isomerase D	Hsp70 protein binding	PMF, MS/MS	3	6.77/40.76	83	3.17
24	P16422	Epithelial cell adhesion molecule	Protein complex binding	PMF		7.42/34.93	131	2.51
25	P37837	Transaldolase	Monosaccharide binding	PMF, MS/MS	4	6.36/37.54	89	2.04
26	P11926	Ornithine decarboxylase	Ornithine decarboxylase activity	PMF		5.10/51.15	79	3.23
27	P09525	Annexin A4	Calcium ion binding	PMF, MS/MS	2	5.83/35.88	169	4.24
28	P06744	Glucose-6-phosphate isomerase	Glucose-6-phosphate isomerase activity	PMF, MS/MS	4	8.42/63.15	213	3.78
29	P27797	Calreticulin	Calcium ion binding	PMF, MS/MS	4	4.29/48.14	228	2.22
30	P05783	Keratin, type I cytoskeletal 18	Scaffold protein binding	PMF, MS/MS	11	5.34/48.06	142	2.13
Proteins with decreasing spot intensity								
31	P30101	Protein disulfide-isomerase A3	Cysteine-type endopeptidase activity	PMF, MS/MS	7	5.98/56.78	103	0.32
32	P28070	Proteasome subunit beta type-4	Threonine-type endopeptidase activity	PMF, MS/MS	2	5.70/29.20	187	0.23
33	Q99LB4	Capping protein (Actin filament), gelsolin-like	Cell projection assembly	PMF		6.47/38.77	109	0.29
34	Q9NNW7	Thioredoxin reductase 2, mitochondrial	Thioredoxin-disulfide reductase activity	PMF		7.24/56.51	93	0.43
35	O95782	AP-2 complex subunit alpha-1	Protein transporter activity	PMF, MS/MS	7	6.63/107.5	167	0.41
36	Q16082	Heat shock protein beta-2	Enzyme activator activity	PMF		5.07/20.23	102	0.38 (0.52)*
37	P42330	Aldo-keto reductase family 1 member C3	Androsterone dehydrogenase activity	PMF		8.06/36.85	146	0.45
38	Q16553	Lymphocyte antigen 6E	Epinephrine secretion	PMF		8.06/13.51	129	0.48
39	P35232	Prohibitin	Sequence-specific DNA binding RNA polymerase II transcription factor activity	PMF, MS/MS	2	5.57/29.80	114	0.28
40	Q92729	Receptor-type tyrosine-protein phosphatase U	Beta-catenin binding	PMF		6.46/16.24	92	0.21
41	P02649	apolipoprotein E	Antioxidant activity	PMF		5.65/36.15	83	0.31
42	Q15181	Inorganic pyrophosphatase	Inorganic diphosphatase activity	PMF, MS/MS	4	5.54/32.66	162	0.25
43	P06576	ATP synthase subunit beta, mitochondrial	Proton-transporting ATP synthase activity, rotational mechanism	PMF, MS/MS	7	5.26/56.56	127	0.41
44	P52597	Heterogeneous nuclear ribonucleoprotein F	RNA binding	PMF, MS/MS	3	5.37/45.67	215	0.46

Note: *The data in the brackets represent the diffenrential ratio obtained from Western blot analysis.

### Western blot confirmation of some of results from proteomics analysis

**Figure 4:**
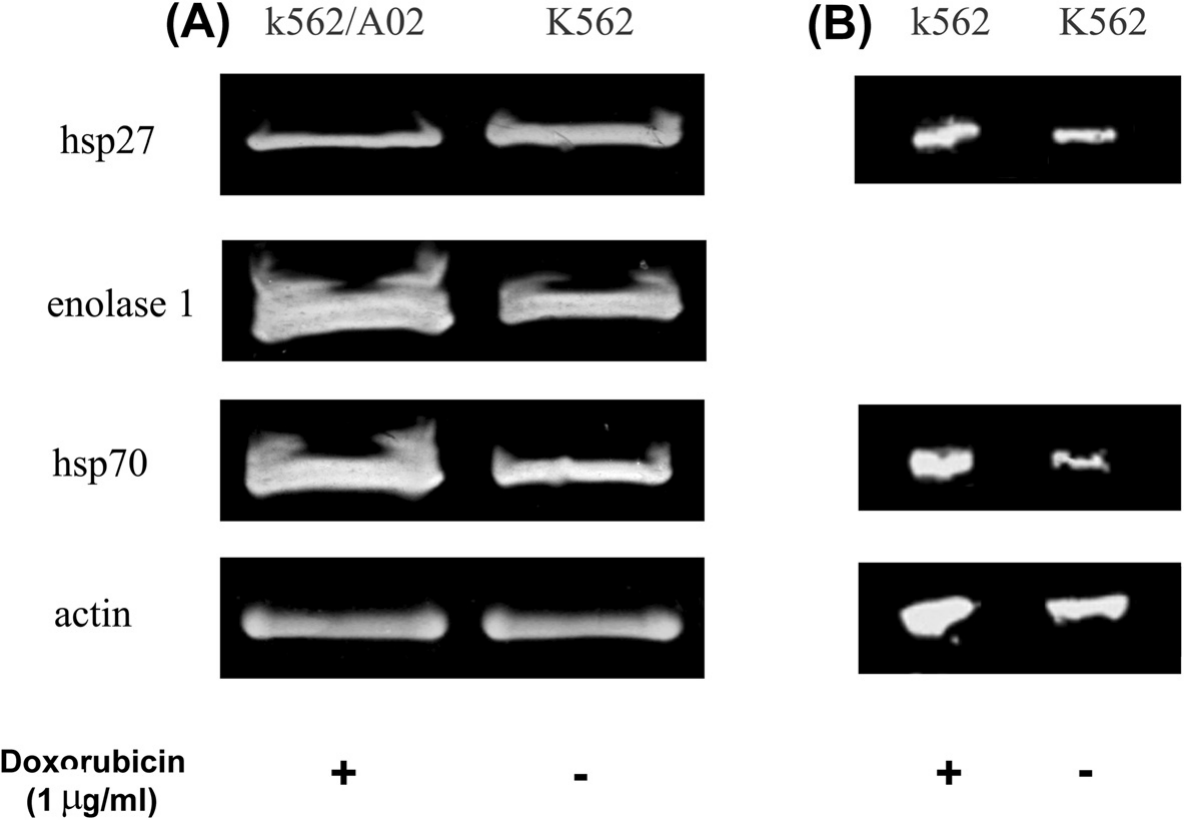
**Western blotting analysis of some of differentially expressed proteins. (A)** Western blotting analysis of HSP70, HSP27, and enolase 1 between K562 cells and K562/A02 cells. The amount of total protein loaded was 20 μg per lane. Differential expression of the protein of interest between doxorubicin-resistant myelogenous leukemia cell line K562/A02 and its parental control cell line K562 is normalized to actin by probing the same membrane with a monoclonal antibody specific for the protein. **(B)** Western blotting analysis of HSP70 and HSP27 in K562 cells with and without 1 microgram/ml doxorubicin treatment for 2 hrs, respectively.

From the identified candidates, HSP70, HSP27 and enolase 1 were selected for western blot analysis as shown in Figure 4. The expression changes of the three selected proteins were consistent with 2-DE results when normalized to actin level. HSP70 and enolase 1 were up-regulated, while HSP27 were down-regulated in K562/A02 cells compared with the parental K562 cells. All experiments were performed for at least three biological replicates. The results of elevated expressions of both HSP70 and enolase 1 were consistent with those from a previous study by Zou L, et al., in which a series of leukaemia-associated antigens in chronic myeloid leukaemia were detected by sera of patients with CML [18]. In addition, We performed Western blotting analysis of both HSP27 and HSP70 for K562 cells exposed to 1 microgram/ml doxorubicin for 2 hrs. In contrast to decreased expression in K562/A02 cells, HSP27 was up-regulated upon drug treatment in K562 cells. For HSP70, elevated expression level was also observed in K562 cells upon drug treatment, of which the fold change was much bigger than that from our proteomics data when expression data of HSP70 in K562/A02 and K562 cells with and without doxorubicin treatment, respectively, were compared.

## Discussion

Among the identified proteins with differently expressed abundances, some of the key enzymes involved in carbohydrate and energy metabolism were significantly up-regulated, implying that much more energy has to be recruited through glycolysis to help cancer cells survive when exposed to chemical stress. The other interesting finding from the identified protein list is that several kinds of proteins that are involved in intracellular ion regulation were identified, including sorcin, protein S100-A4, annexin A4, calreticulin, chloride intracellular channel protein 1, etc., indicating that metal ions such as calcium might play a pivotal role in the development of chemoresistance.

### Proteins involved in carbohydrate and energy metabolism

Early in 1956, Warburg reported that normal cells derive most of their energy though the Krebs cycle (aerobically), whereas cancer cells derive most of their energy from glycolysis (anaerobically). Over the past few years, several studies published in leading scientific journals showed that an increased rate of glycolysis maintained by tumor cells [19] may possibly be due to altered expression of enzymes and as a result, tumor cells burn much more glucose for energy than normal cells [20,21].

In our study, two enzymes involved in glycolysis, namely fructose-bisphosphate aldolase A, fructose-bisphosphate aldolase C, transaldolase and alpha-enolase, were found to be up-regulated in doxorubicin-resistant myelogenous leukemia cells. It was worthy of note that one of the enzymes involved in glycolysis process, namely enolase, was dramatically up-regulated in doxorubicin-resistant myelogenous leukemia cells. Enolases are glycolytic enzymes that interconvert 2-phosphoglycerate to phosphoenolpyruvate. in glycolysis, by which low-energy phosphate ester bond of 2-phosphoglycerate is converted into the high-energy phosphate bond of PEP. In a study by Tu SH and his co-workers [22], alpha-enolase was found to be up-regulated in both tamoxifen-resistant breast cancer and head-and-neck cancer. Additionally, both chemoresistance and invasive ability of these tumors can be dramatically suppressed through knockdown of alpha-enolase expression, implying that up-regulation of enolase 1 may have a protective effect against chemical pressure by augmenting anaerobic metabolism.

The other up-regulated enzyme involved in glycolysis found in doxorubicin-resistant myelogenous leukemia cells, aldolase, splits fructose 1,6-biphosphate into two three-carbon molecules, glyceraldehydes 3-phosphate and dihydroxyacetone phosphate, in glycolysis. Glyceraldehyde 3-phosphate is the only molecule that can be used for the rest of glycolysis. However, the dihydroxyacetone phosphate can be converted to glyceraldehyde 3-phosphate by triose phosphate isomerase. Unfortunately, the evidence of the direct relationship between aldolase and chemoresistance in tumor cells has been poorly documented and the role of aldolase in development of chemoresistance in doxorubicin-resistant myelogenous leukemia cells needs to be further investigated.

Our findings suggest that elevated level of enolase 1 and aldolase C may contribute directly or indirectly to the chemoresistance demonstrated by doxorubicin-resistant myelogenous leukemia cells and the cells need more energy through glycolysis to survive when exposed to chemical stress. Therefore, oncologists should pay much attention on glycolysis as a major target area for the development of new strategies to overcome chemoresistance in cancer patients [23,24].

### Proteins involved in intracellular ion regulation

Accumulated evidences have suggested that intracellular calcium may play a role in the development of chemoresistance in some cell lines [25,26]. In our present study, several calcium-binding proteins were identified in doxorubicin-resistant myelogenous leukemia cells, such as sorcin, protein S100-A4, annexin A4, calreticulin, etc., indicating that metal ions such as calcium might play a pivotal role in the development of chemoresistance. A soluble cytosolic calcium-binding protein, sorcin, has been reported to be implicated in development of resistance to cytotoxic chemicals [27,28], although its contribution to multi-drug resistance remains uncertain. Protein S100-A4 belongs to a member of the S100 calcium-binding protein family, and it was observed to be up-regulated in doxorubicin-resistant myelogenous leukemia cells. Similar results were obtained from a recent study by Yang M et al., in which elevated expression level of S100-A8 was detected in drug resistance leukemia cell lines relative to their drug sensitive cell lines. Further studies indicated that S100A8 might contribute to drug resistance in leukemia by regulating autophagy [29]. Interestingly, another member of the S100 calcium-binding protein family, S100P, was reported to contribute to chemosensitivity in gastric cell lines by increasing drug inflow [30]. Our data suggested that S100-A4 may be a novel target for improving leukemia therapy.

### Proteins that function in cell motility or structure

Besides the mechanical role in cell integrity [31,32], cytoskeletal keratins (CKs) also perform important functions in cellular defense mechanism in response to various stresses, including chemical agents. Several studies have shown that both sumoylation and phosphorylation of CKs were raised under cell stress [33,34]. In addition, hyperphosphorylation of CK 8 at Ser-73 was implicated in apoptosis induced by anisomycin or etoposide in cultured HT-29 cells [35]. Tao He, et al., suggested that Ser-73 could be a putative target for the stress-activated MAPK family members [36]. Besides the regulatory role of phosphorylation of CKs in cell signaling pathway under stress, notably, from an energetic point of view, marked overexpression and hyperphosphorylation of CK 8 and CK 18 may support cells in handling several forms of stress that may lead to cell death, because phosphorylation of CKs reduces intracellular ATP levels and may help maintain a phosphate reserve.

Vimentin, another kind of intermediate filament proteins mainly expressed in cells originating from mesenchyme, is thought to play a key role in cell growth, cell cycling and cell resistance to mechanical or chemical stress. In contrast to keratins, vimentin tends to assemble homopolymeric filaments. Belichenko I [37] suggested that the full-length vimentin may be implicated in survival signaling and confers resistance to nuclear apoptosis after photodynamic treatment. Significant elevation of vimentin expression during the process of tumorgenesis was reported in recent years [38,39]. In principal, our data suggest that dramatically increased expression level of vimentin in doxorubicin-resistant myelogenous leukemia cells contributes to the resistance to cytotoxic agent to some extent.

### Group of molecular chaperones

It is well known that heat shock and other chemical stresses stimulate synthesis of a specific group of proteins called heat shock proteins (HSPs). These proteins are implicated in cellular protection mechanisms and play an important role in protection of proteins from denaturation by stress-inducing agents and the repair or degradation of polypeptides that have been denatured under stresses in an ATP-dependent manner [40,41]. The increased expression of several HSPs was very common in most chemoresistant variants. However, in the present report, only the HSP70 was found to be up-regulated significantly. An unexpected decrease in the expression of HSP27 was reproducibly observed in the doxorubicin-resistant myelogenous leukemia cells. These results suggest that molecular chaperones, such as HSP27 and HSP70, etc., may execute protective function in systematically different ways in doxorubicin-resistant myelogenous leukemia cells.

To our knowledge, this uncommon expression pattern of HSP27 in doxorubicin-resistant myelogenous leukemia cells has not been previously described. Unlike HSP70, known be implicated in cell proliferation, HSP27 may be involved in cell growth arrest and increased differentiation [42,43]. Several lines of evidence have been clearly indicated that HSP27 is involved in the regulation of actin polymerization [44,45]. In doxorubicin-resistant myelogenous leukemia cells, the decrease of expression level of HSP27 and actin filament (also termed capping protein) revealed in our experiments implied that down-regulation of these proteins may be indirectly implicated in the cell resistance against cytotoxic agents by retarding or reducing differentiation events. In principal, however, the down-regulation of the these proteins in doxorubicin-resistant myelogenous leukemia cells is intriguing and further studies will be required to elucidate the mechanism underlying the reduced expression level of them in this drug resistant cell line.

### Proteins that are related to oxidation or reduction

Another interesting finding is the involvement of protective proteins, peroxiredoxins, in the development of drug resistance the chemoresistant cell line K562/A02. Besides playing a role as a peroxidase, these protective proteins have been suggested to serve multiple functions involved in various biological processes such as the detoxification of oxidants, cell differentiation and intracellular signaling. Non-selenium glutathione peroxidase (also termed peroxiredoxin 6 or antioxidant protein 2) is a member of family of antioxidant proteins [46]. Unlike other members in the family of anti-oxidative proteins, nonselenium glutathione peroxidase contains only one Cys residue and has been reported to exhibit glutathione peroxidase and phospholipase A2 activities [47]. A body of evidence has accumulated to suggest that protein glutathionylation may be a mechanism of redox regulation of protein functions. A variety of proteins, such as enzymes, transcription factors, and oncogenes, have been reported to be glutathionylated [48,49]. Further studies would lead to a better understanding of biological functions of members of the peroxiredoxin family in doxorubicin-resistant myelogenous leukemia cells.

Protein disulfide isomerase, a primary folding catalyst and chaperone located in the endoplasmic reticulum (ER), is involved in rearrangement of disulfide bridges in proteins to form the correct protein structures. In contrast to HSPs in the cytosol where maintains a more reducing condition than ER, the protein disulphide isomerase turns to act as a chaperone primarily when reduced [50]. However, information on the role of protein disulphide isomerase in doxorubicin-resistant myelogenous leukemia cells is poorly documented and down-regulation of it seems that processes involved in endoplasmic reticulum may be quite different with those in cytosol.

## Conclusions

As a whole, under the chemical stress, the doxorubicin-resistant myelogenous leukemia cells may employ various protective strategies to survive. These include: (i) pumping the cytotoxic drug out of the cells by P-glycoprotein, (ii) increased storage of fermentable fuel, (iii) sophisticated cellular protection by molecular chaperones, (iv) improved handling of intracellular calcium, (v) increased glucose utilization via increased rates of glycolysis. In the present study, proteomic analysis of leukemia cells and their drug resistant variants revealed multiple alterations in protein expression. Our results indicate that the development of drug resistance in doxorubicin-resistant myelogenous leukemia cells is a complex phenomenon undergoing several mechanisms.

## Methods

### Cell culture

The drug-resistant MDR cell line K562/A02 was obtained from the Institute of Hematology, Tianjin, China. It shows resistance not only to doxorubicin but also to some other structurally unrelated lipophilic cytotoxic drugs, including harringtonine, vincristine, amsacrine, etc. The cells were maintained in RPMI 1640 (GIBCO) supplemented with 10% fetal bovine serum (Hyclone) at 37°C in a humidified 5% CO2 atmosphere. In addition, 1 μg/ml doxorubicin was added into the medium to maintain the drug resistance. The drug-sensitive parental cell line K562 was kept in our laboratory, and cultured in the same condition without doxorubicin.

### Cytotoxicity assay

Cytotoxicity of doxorubicin was measured using the MTT (3-[4, 5-dimethylthiazol-2yl]-2, 5-diphenyl tetrazolium bromide) (MTT; Sigma-Aldrich) assay [51]. Briefly, 2 × 104 cells per well were seeded into 96-well culture plates and kept for 24 h. After doxorubicin exposure for 48 hours, the medium was removed, followed by addition of an equal volume of fresh medium containing 0.5 mg/ml MTT. After the cells were incubated with MTT for 4 h at 37°C, the medium was replaced with 200 μl DMSO and kept for 30 min at room temperature. The absorbance was recorded using a microplate reader at absorption wavelength of 570 nm. The cytotoxic effects of drugs were calculated according to the OD values.

### Sample preparation for 2-DE

Cells were washed three times with a solution containing 10 mM Tris, 1 mM EDTA and 250 mM sucrose which was adjusted to pH 7.0-7.5, and cell lysates were then prepared using lysis buffer (8 M urea, 4% w/v CHAPS, 50 mM DTT, 25 mM spermine, a cocktail of proteinase inhibitors from Roche). After 60 min of gentle stirring at room temp, the sample was centrifuged at 40 000 g for 60 min. The supernatant was then collected and protein concentration was determined using the DC RC protein assay kit (Bio-Rad), following the manufacturer’s instructions. The samples were then aliquoted and stored at −80°C until used for 2-DE.

### 2-DE

Isoelectric focusing was carried out using Protean IEF Cell (Bio-Rad). Samples containing 0.6 mg for semi-preparative gels, were diluted to 300 μl with rehydration solution (6 M urea, 2% w/v CHAPS, 65 mM DTT, trace bromophenol blue), to which either pH 3.9-5.1, 4.7-5.9, 5.5-6.7 or 6.3-8.3 Bio-lyte was added to final concentration of 0.5% v/v. The samples were accordingly applied to IPG gel strips (pH 3.9-5.1, 4.7-5.9, 5.5-6.7 and 6.3-8.3, 17 cm, Bio-Rad) for 14 h in a passive mode. Proteins absorbed into IPG gel strips were focused for 80 kVh at 20°C. After equilibrated in equilibration solution, gel strips were applied on second-dimensional PAGE with 12.5% polyacrylamide. Separation was then carried out on a Protean II xi electrophoresis system (Bio-Rad) at a current setting of 7.5 mA/gel for the initial 1 h and 15 mA/gel until the bromophenol blue reached the bottom of the gel.

### Protein visualization and image analysis

For colloidal Coomassie blue G-250 staining of semi-preparative gels, gels were fixed in 10% TCA for 60 min and rinsed in Milli Q water for 20 min. Gels were then stained in a solution containing 20% methanol, 2% phosphoric acid, 10% ammonium sulfate and 0.1% Coomassie Brilliant Blue G-250 for 24 h. The stained gels were then rinsed in MilliQ water for 20 min to remove any dye residue. Then the stained gels were canned with a high-resolution scanner (Umax 1120) and the gel images were analyzed using PDQuest software (Version 7.1.1; Bio-Rad) according to the protocols provided by the manufacturer. To accurately compare spot quantity between gels, a normalization based on the total density on each gel was applied for each gel and normalized spot intensities were expressed in ppm. Student’s t-test was performed on the replicate gels between the chemoresistant cell line K562/A02 and the parental cell line K562. The significantly differentially expressed protein spots (p < 0.05) with 2-fold increased or decreased intensity between the chemoresistant variant K562/A02 and the parental cell line K562 were selected and subject to further identification by MALDI-TOF and LC-MS/MS.

### MALDI-TOF MS analysis and database searching

Differential spots were excised from semi-preparative gels and transferred to 1.5 ml siliconized Eppendorf tubes. The gel-spots were washed and then destained by 50% ACN until the blue dye turned invisible. After dried in a vacuum centrifuge, the gel-pieces were incubated in the digestion solution containing 50 mmol/L NH4HCO3 and 0.1 g/L TPCK-trypsin for 12 h at 37°C. The resulting peptides were extracted three times by 50 μl aliquots of 5% trifluoroacetic acid in 60% acetonitrile. Combined extracts were concentrated in a Speed Vac to 3–5 μl. The concentrated tryptic peptide mixture was mixed with saturated CHCA matrix solution and vibrated gently. A volume (1 μl) of the mixture containing CHCA matrix was loaded on a 96 × 2 well hydrophobic plastic surface sample plate (Applied Biosystems) and air-dried. The samples were analyzed with Voyager DE STR MALDI TOF Mass Spectrometer (Applied Biosystems). A peptides mixture containing Angiotensin I, ACTH (1–17) and ACTH (18–39) was used as mass standards for external calibration. Monoisotopic peak masses were used to search against the Swiss-Prot database using MASCOT search engine with the following parameters: one missing cleavage, peptide tolerance of 100 ppm, variable methionine oxidation and fixed cysteine carbamidomethylation.

### Nano-ESI-MS/MS analysis and database searching

The lyophilized tryptic peptides were redissolved in 0.1% formic acid and then desalted by using ZipTip C18 pipette tips (Millipore, Bedford, MA, USA). The samples were loaded into a nanoelectrospray needle and analyzed on a quadrupole orthogonal acceleration TOF mass spectrometer (QSTAR, Applied Biosystems) equipped with an external nanoelectrospray ion source. Once the full mass spectra (parent ions) of the samples were obtained in TOF MS mode, MS/MS experiments for individual peptide ions with doubly- or triply-charge state were performed in Product ion mode. The data from each sample were searched against the Swiss-Prot database on a local MASCOT server (version 2.1, Matrix Science) using a script embedded in the ProteinPilot 4.5 software (MDS Sciex, South San Francisco, CA, USA). Following parameters were set during database searching: one missed cleavage; peptide tolerance, 0.2 Da; MS/MS tolerance, 0.2 Da; cysteine carbamidomethylation as fixed modification and methionine oxidation as variable modification.

### 1-DE and western blot analysis

Samples containing equivalent amounts of protein in SDS loading buffer were subjected to electrophoresis in 12% Tris-glycine-SDS polyacrylamide gel using a Mini-Cell system (Bio Rad). After electrophoresis, proteins were electroblotted to polyvinylidene fluoride (PVDF) membranes (Millipore). After blocked with 1.5% nonfat dried milk in TBST (25 mM Tris, pH 7.5, 150 mM NaCl, 0.05% Tween 20, and 0.001% thimerosal) for 1 h at room temperature, membranes were incubated with primary antibody at room temperature for 2 h. Several primary antibodies were chosen: mouse anti-human Hsp70 (Santa Cruz Biotechnology, used at 1:250 dilution), goat anti-human enolase 1 (Santa Cruz Biotechnology, used at 1:225 dilution), mouse anti-human actin (Santa Cruz Biotechnology, used at 1:250 dilution). After washed 10 min in TBST solution, membranes were incubated with properly diluted secondary antibody conjugated with horseradish peroxidase for 1 hr at room temperature. Membranes were washed again and stained with 0.05% diaminobenzidine in Tris–HCl (100 mM, pH 7.5) containing 0.035% hydrogen peroxide.

## Additional file

Additional file 1: **Supplementary materials.**

## Abbreviations

CML: Chronic myelogenous leukemia
2DE: Two dimensional electrophoresis
IEF: Isoelectric focusing
HSP: Heat shock protein
MALDI-TOF: Matrix-assisted laser desorption/ionization-time of flight
AML: Acute myelogenous leukemia
MDR: Multi-drug resistance
Pgp: P-glycoprotein
IPGs: Immobilized pH gradients
MS: Mass spectrometry
CKs: Cytoskeletal keratins
ER: Endoplasmic reticulum
