# Supplementary material for: Comparative proteomics analysis of differential proteins in respond to doxorubicin resistance in myelogenous leukemia cell lines
Source: Proteome Sci. 2015 Jan 22;13:1. doi: 10.1186/s12953-014-0057-y (PMC4307195; doi:10.1186/s12953-014-0057-y)
Supplement: Supplementary file 1 — Supplementary materials. [file 12953_2014_57_MOESM1_ESM.doc]

**MS ID:  Proteome Science, 1142149502137008
MS TITLE:  Comparative proteomics analysis of differential proteins in respond to doxorubicin resistance in myelogenous leukemia cell lines**

**Supplementary materials**

**Corresponding authors:**

Chengyan He, Jilin University China-Japan Union Hospital, Changchun 130033, China. E-mail: chengyanhe469@vip.sina.com

Heat shock 70 kDa protein 1A/1B


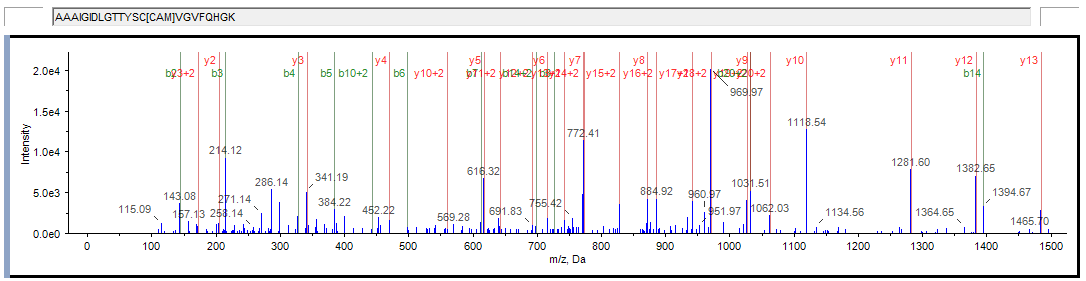


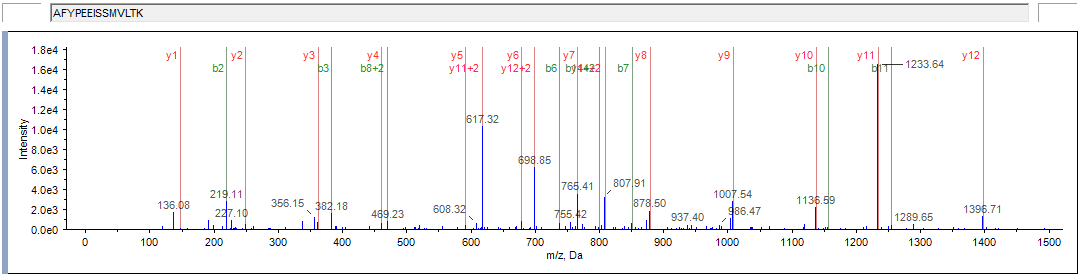


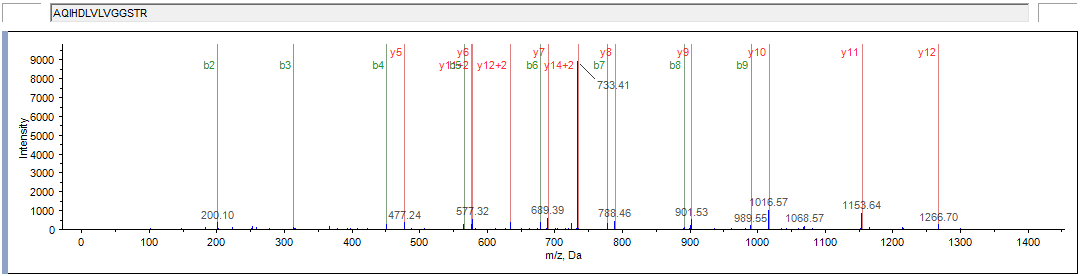


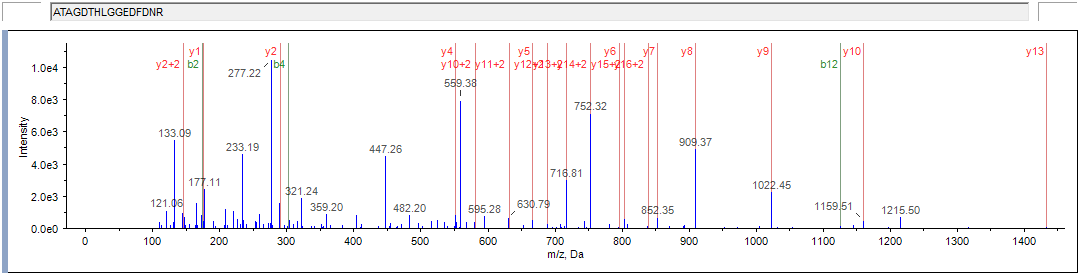


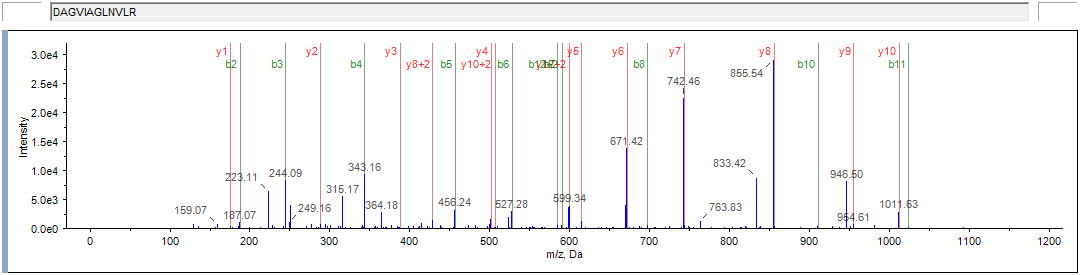


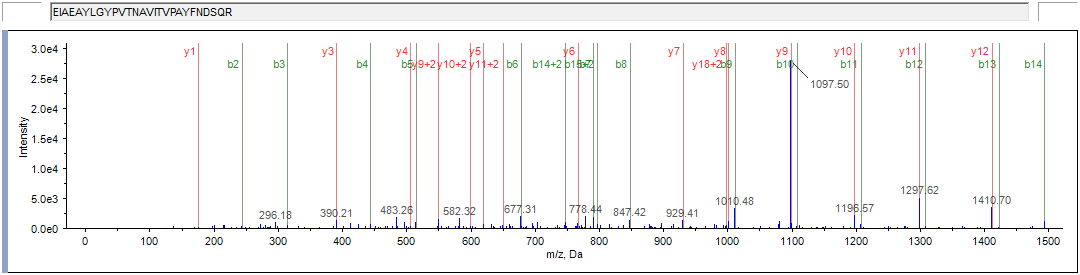


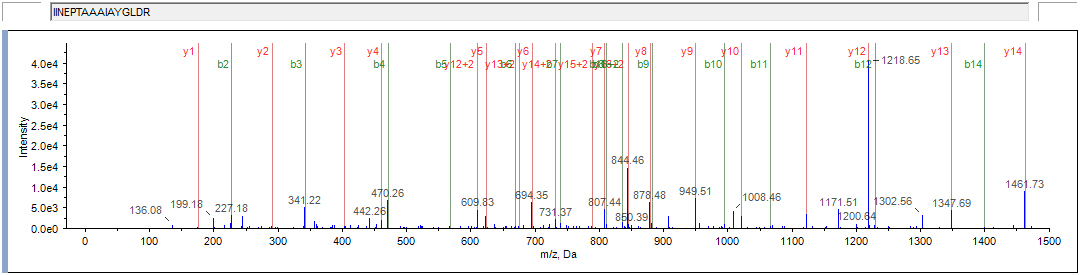


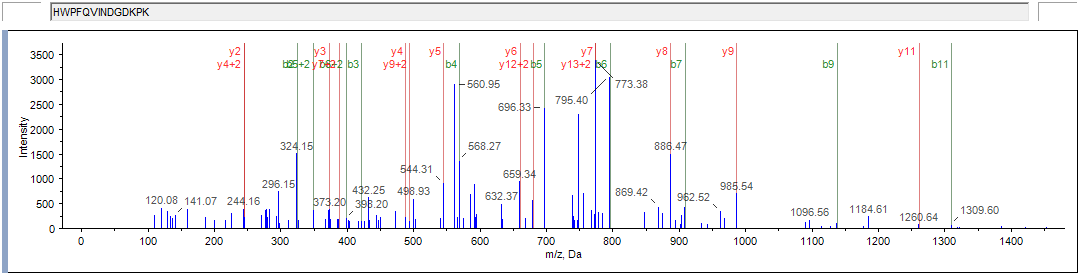


T-complex protein 1 subunit alpha

17


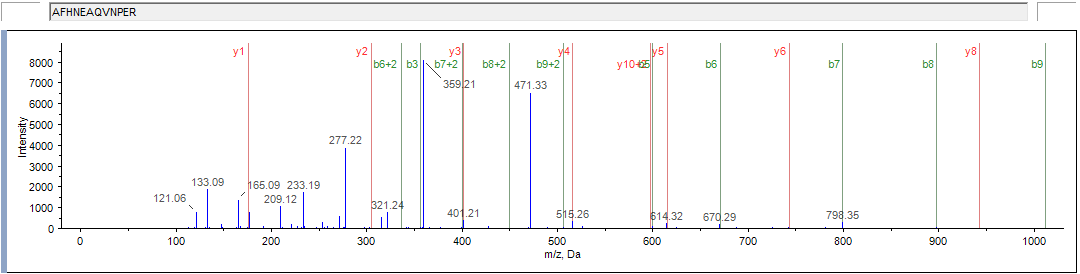


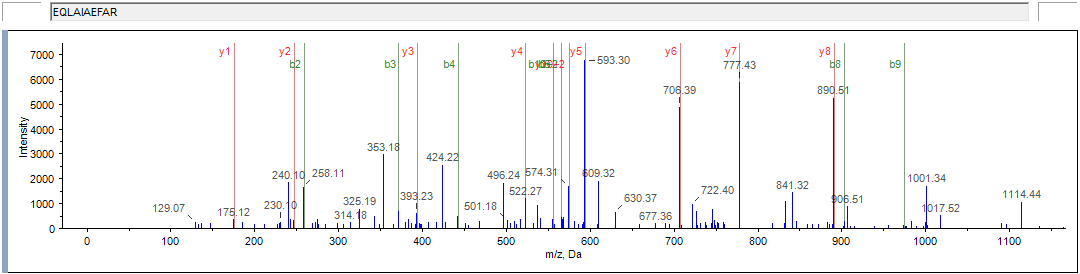


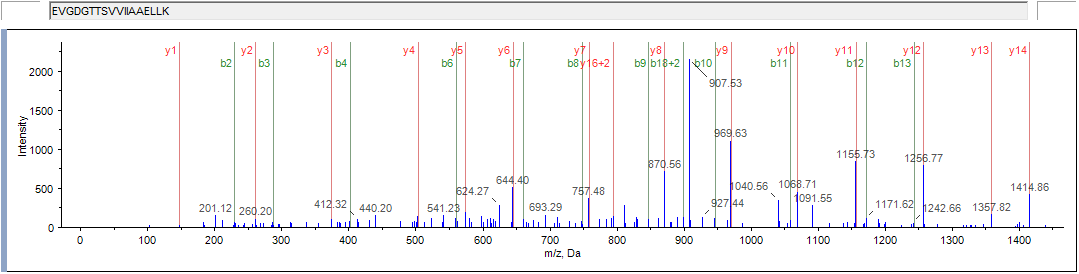


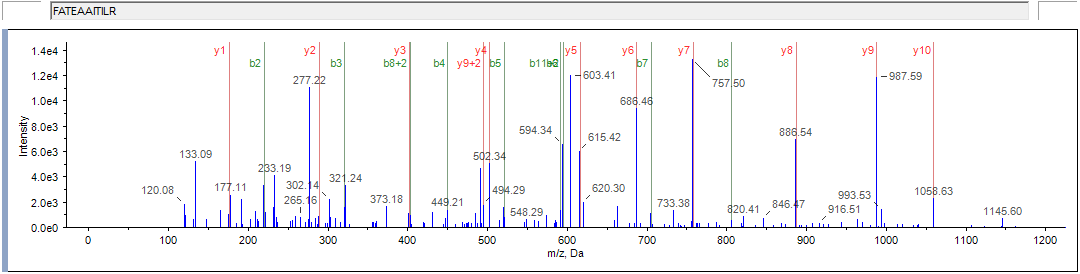


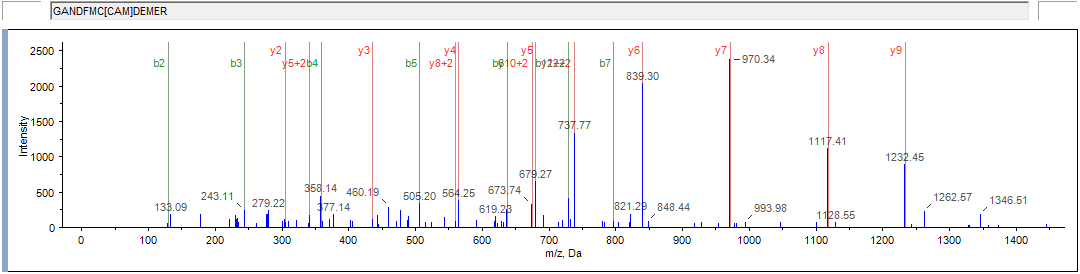


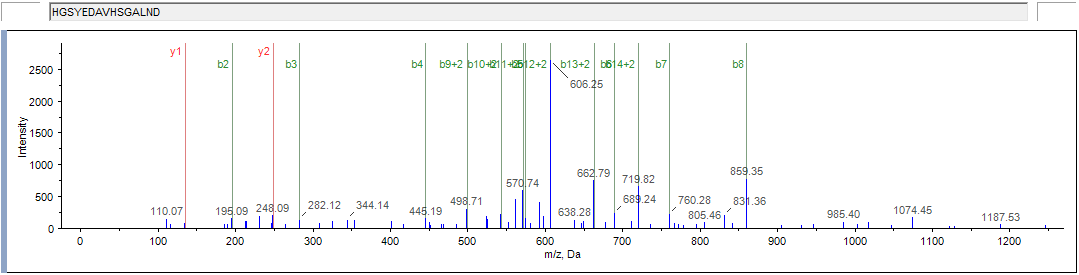


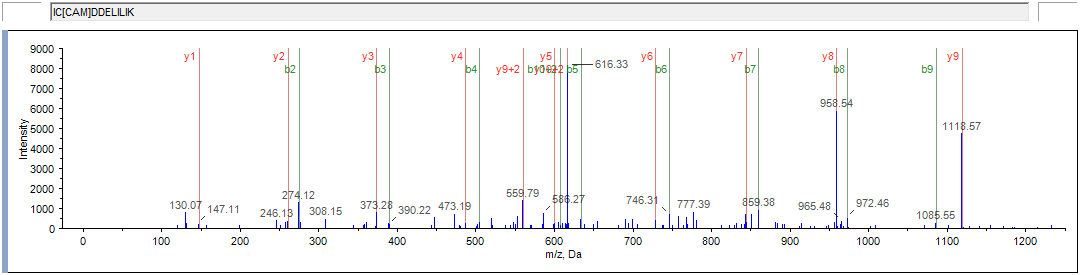


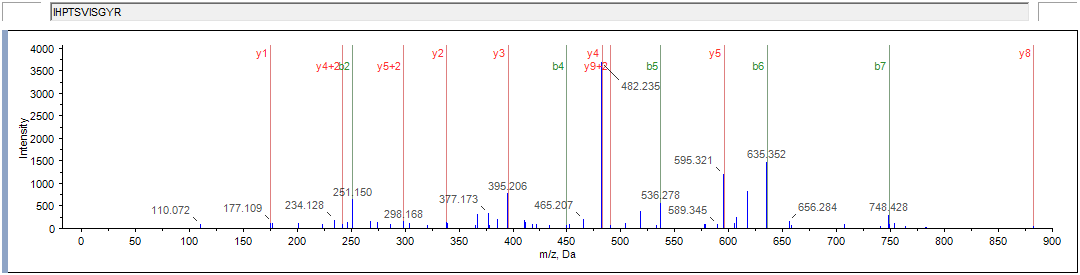


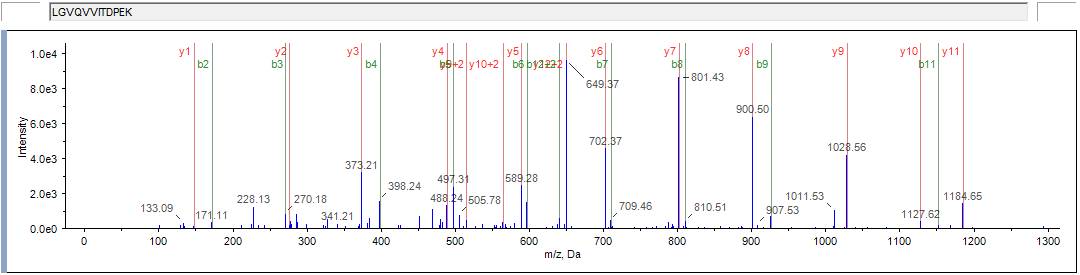


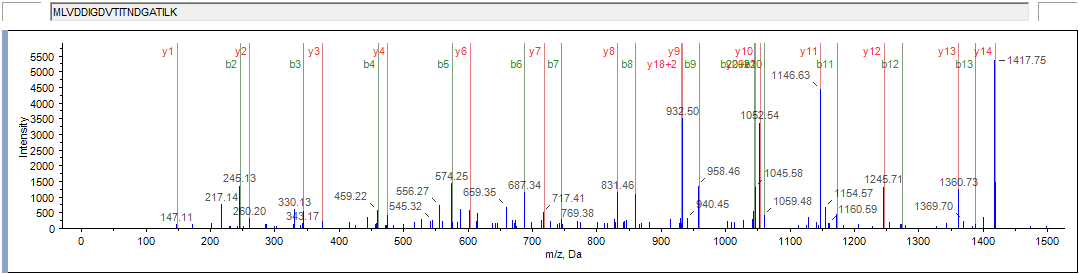


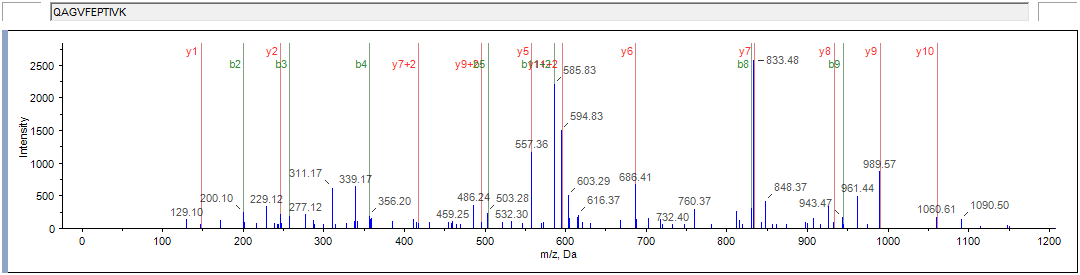


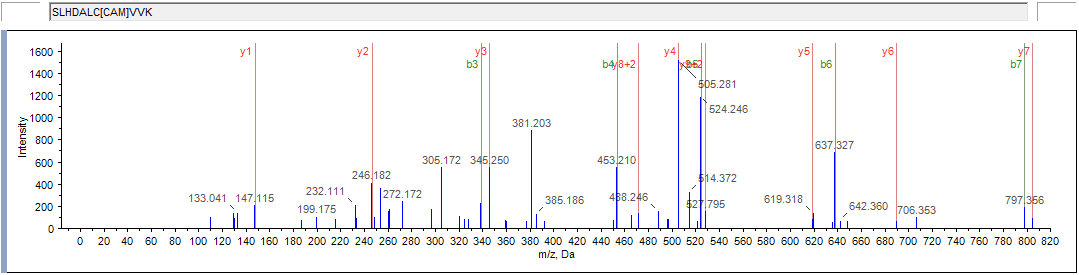


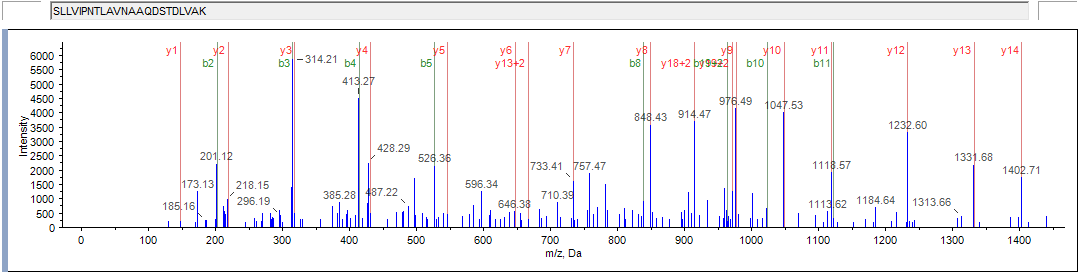


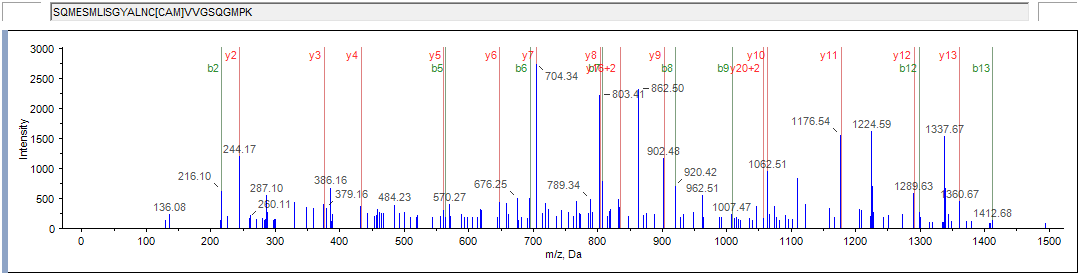


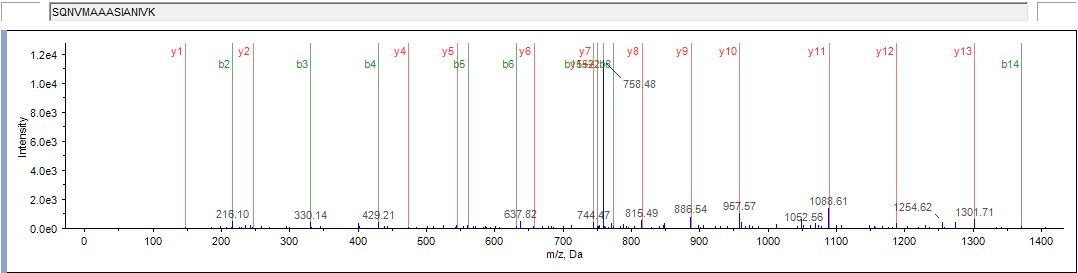


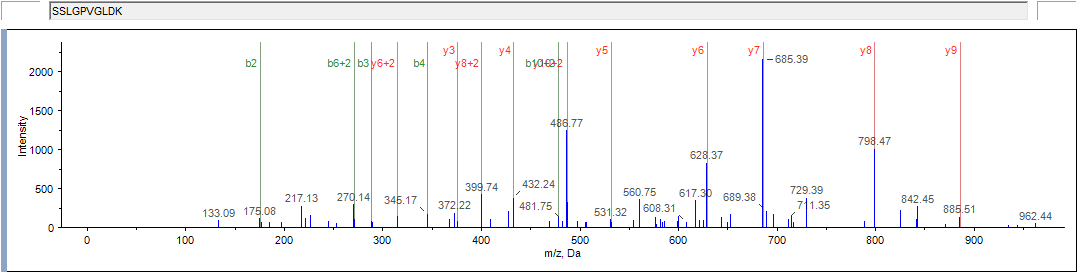


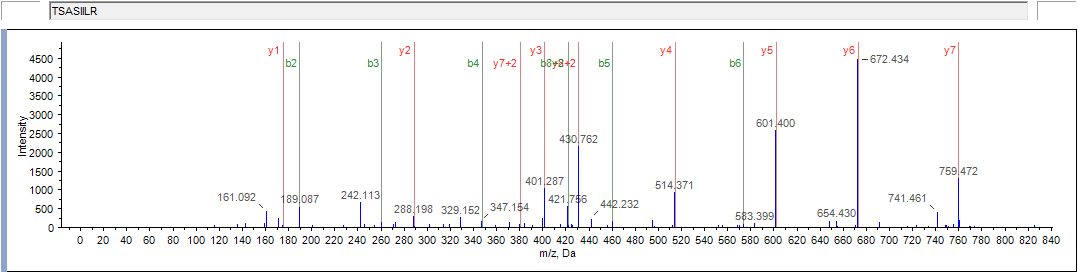


Serpin B9

4


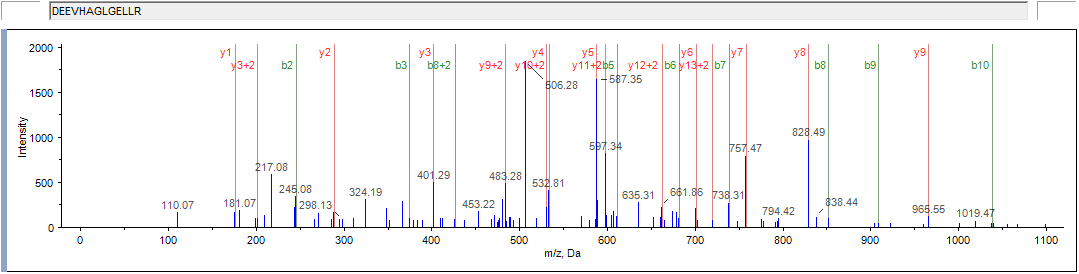


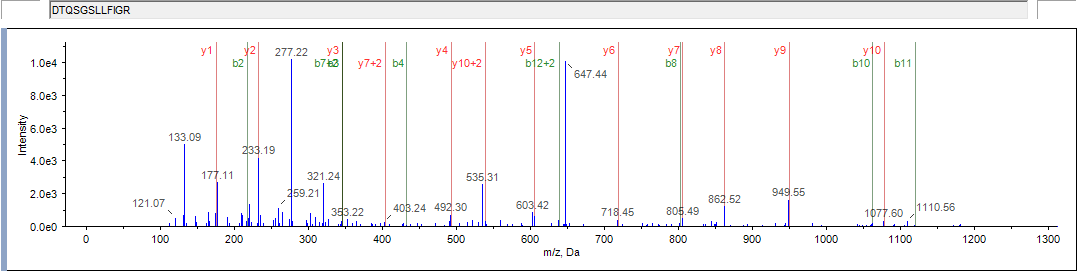


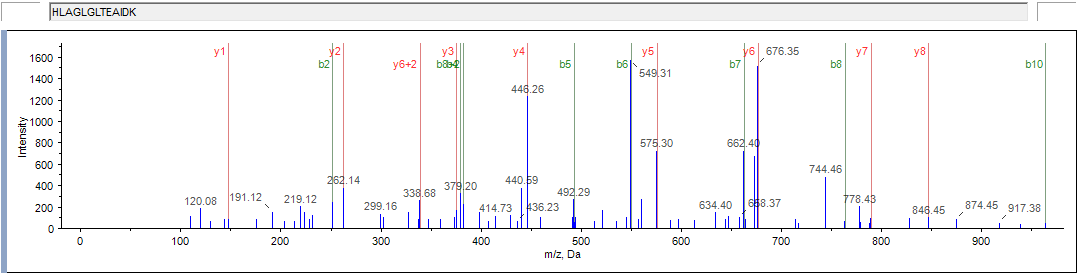


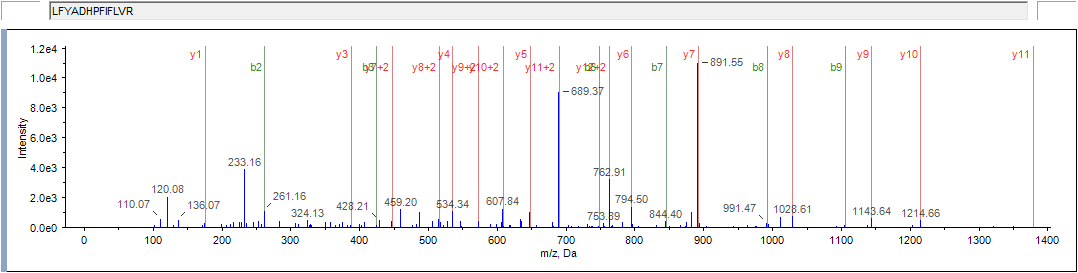


Inosine-5'-monophosphate dehydrogenase 1

5


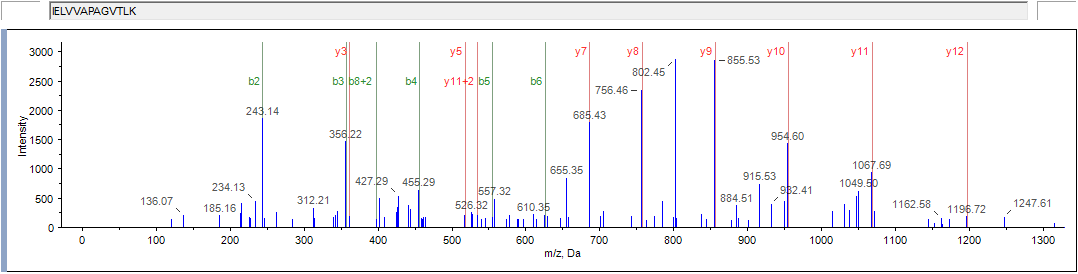


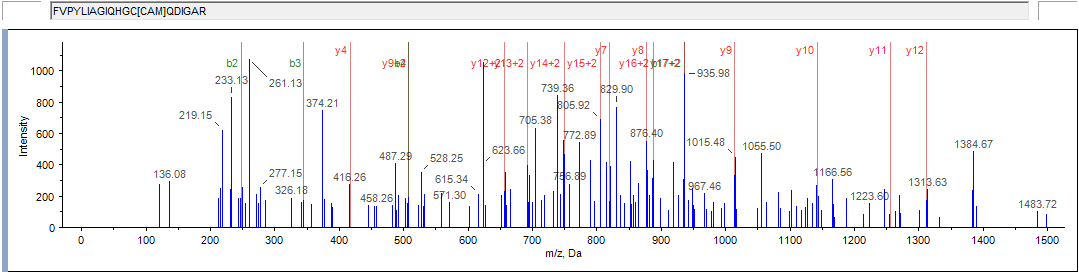


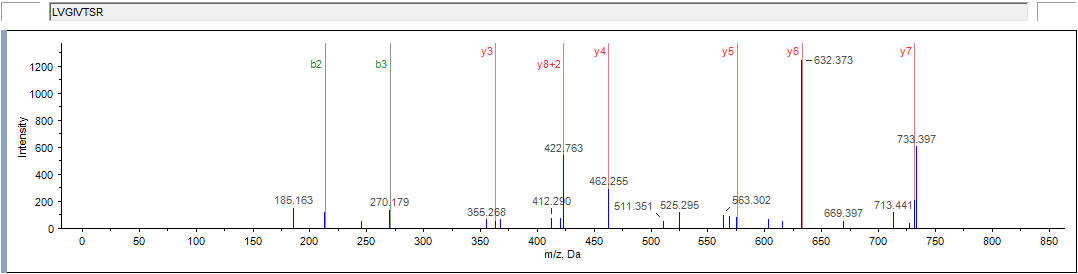


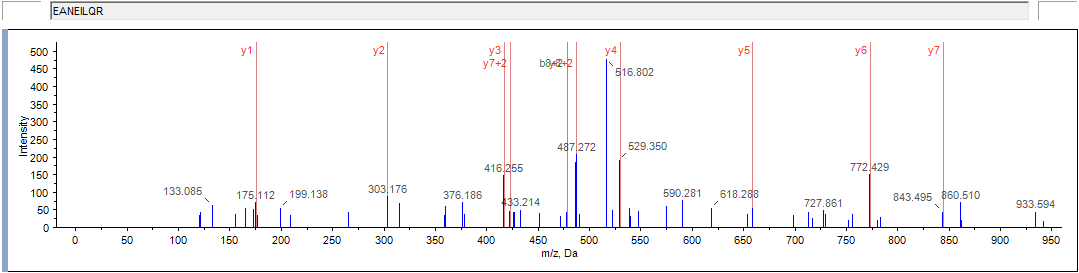


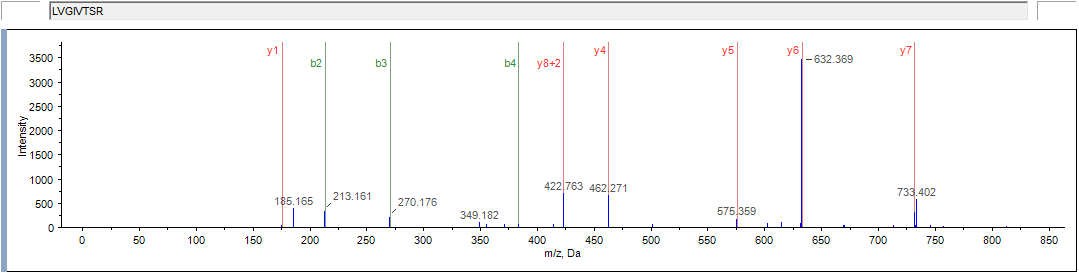


Creatine kinase U-type


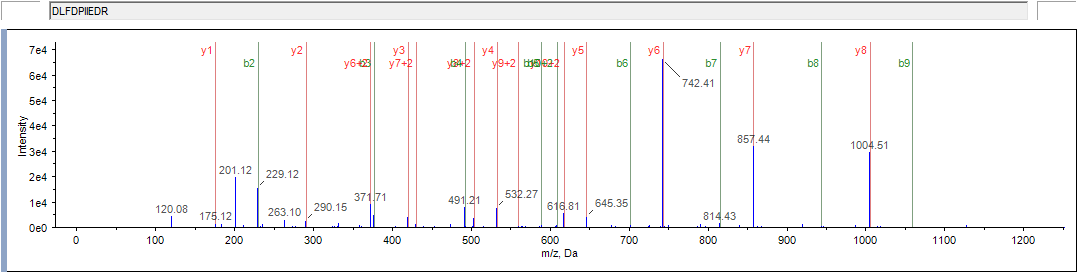


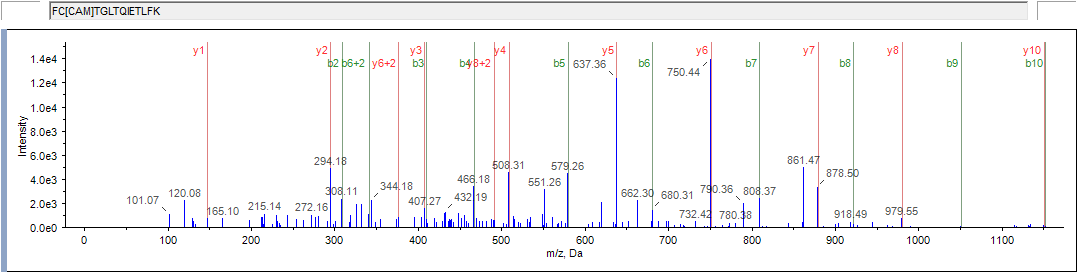


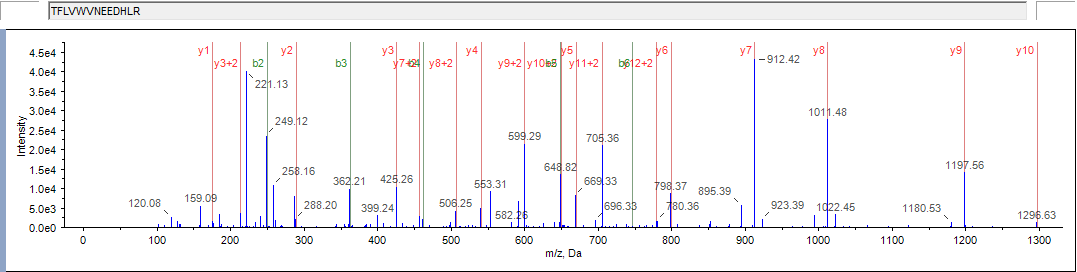


Rho GDP-dissociation inhibitor 1

5


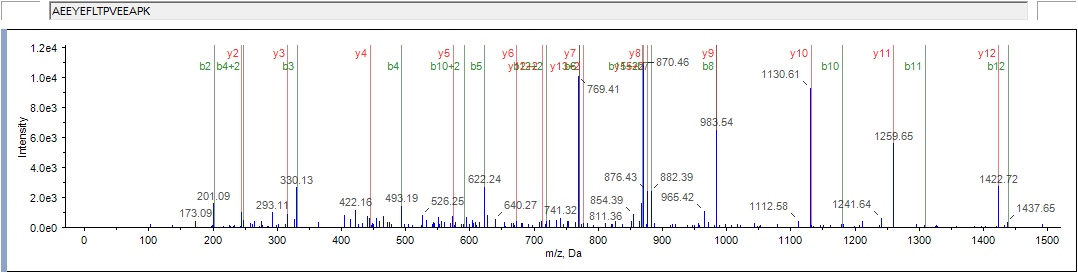


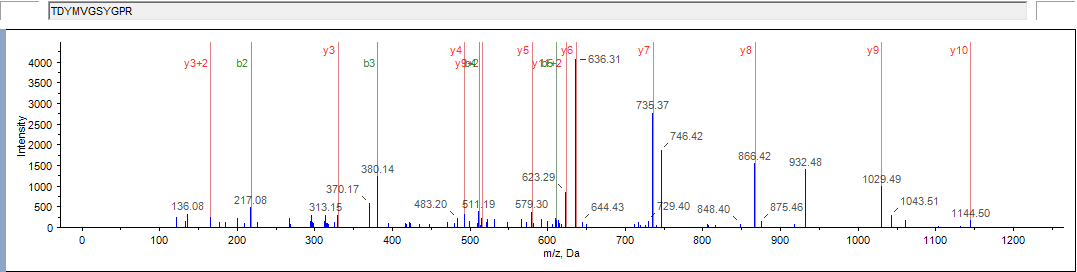


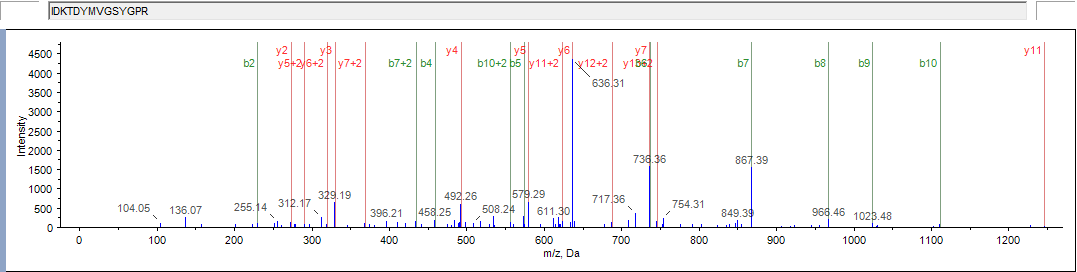


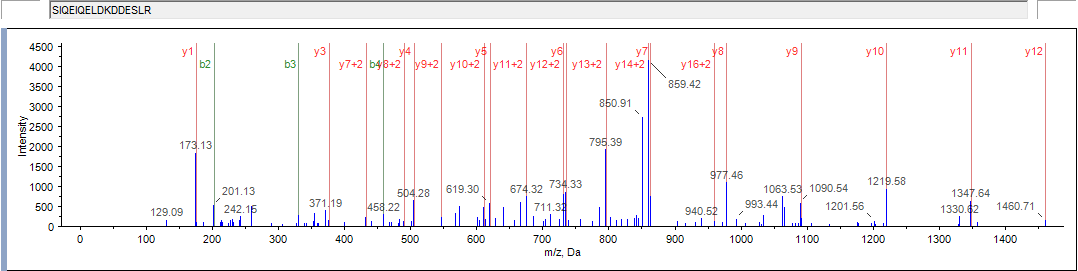


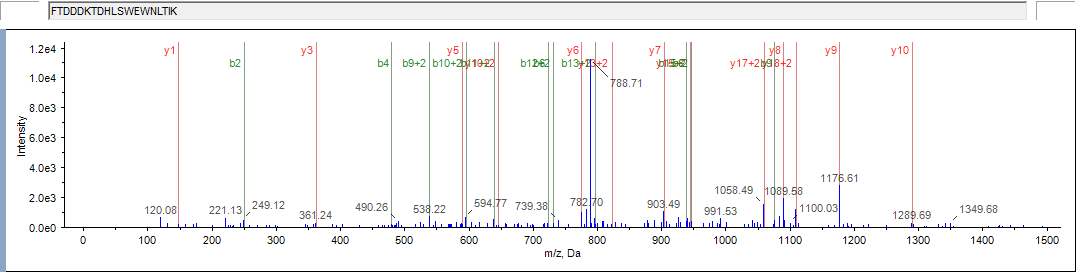


Peroxiredoxin-6

8


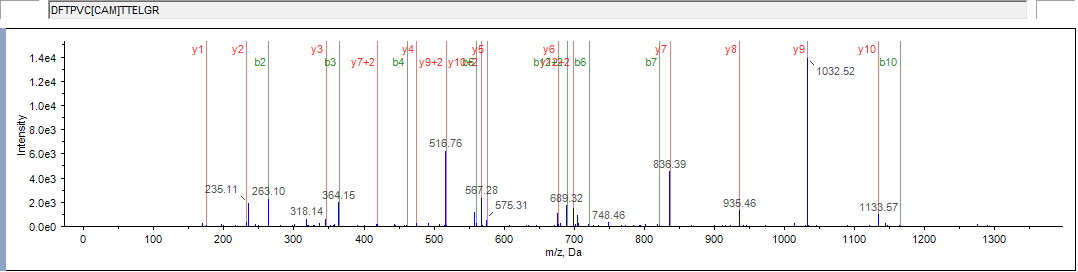


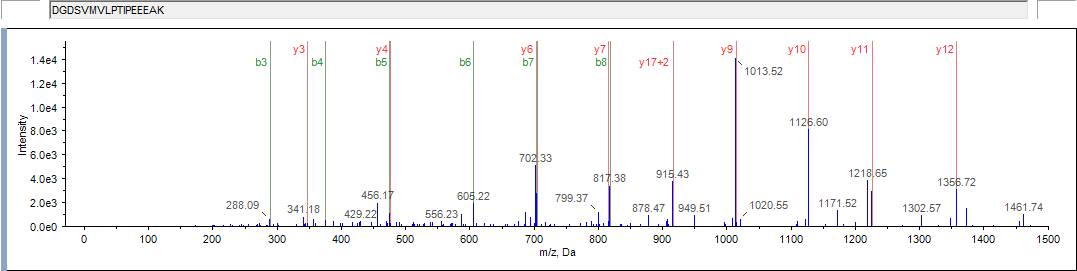


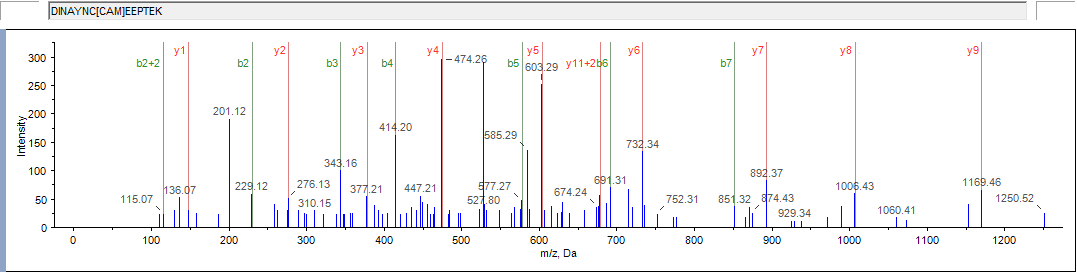


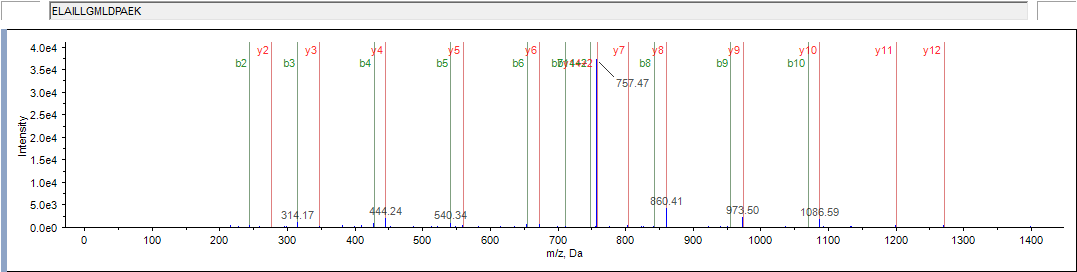


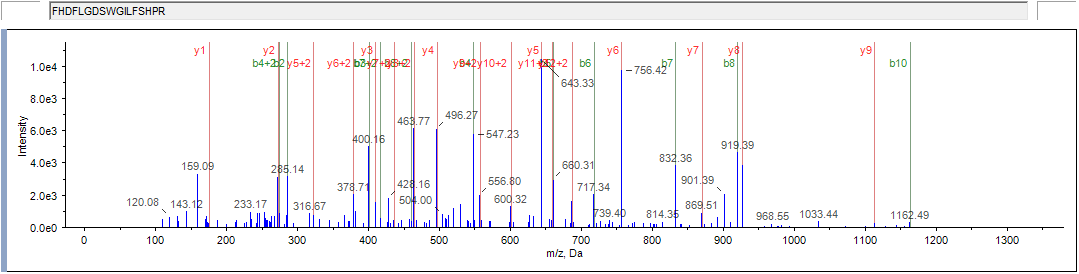


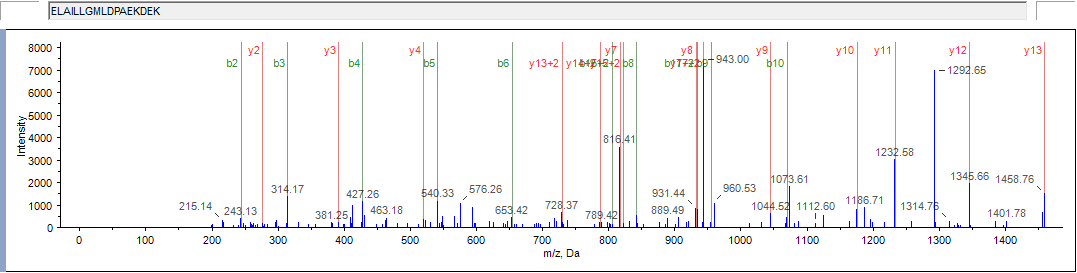


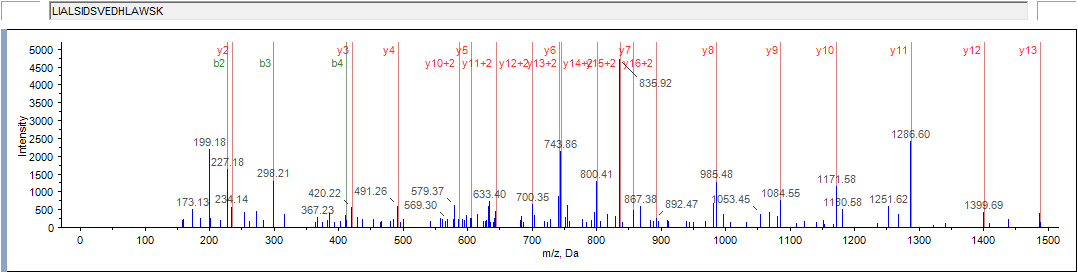


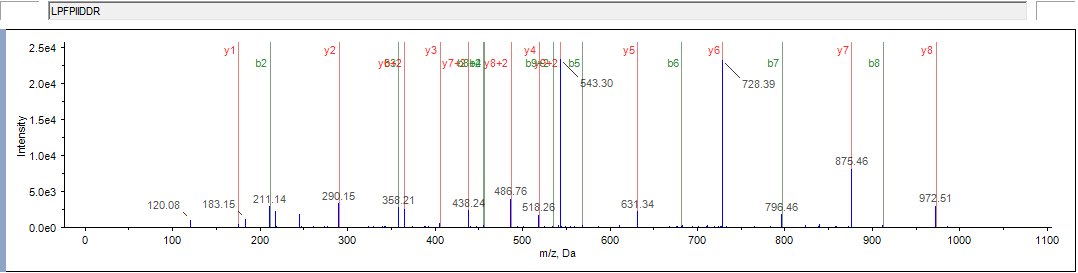


Chloride intracellular channel protein 1

9


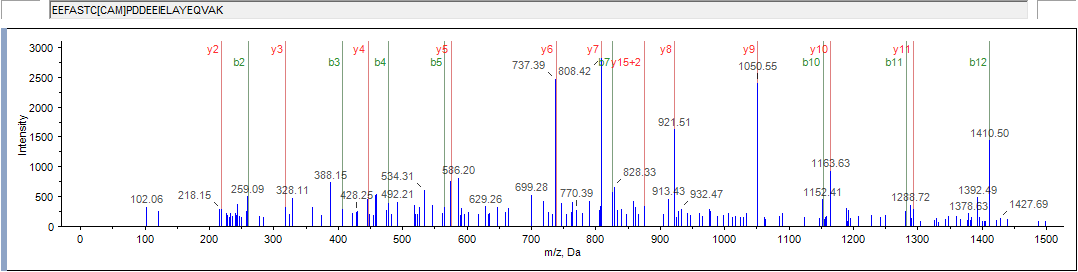


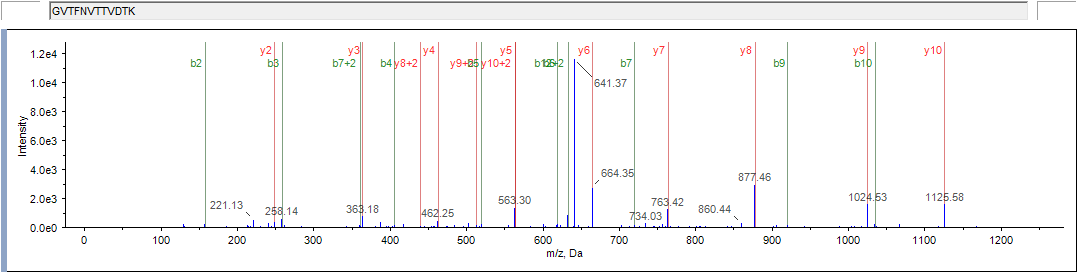


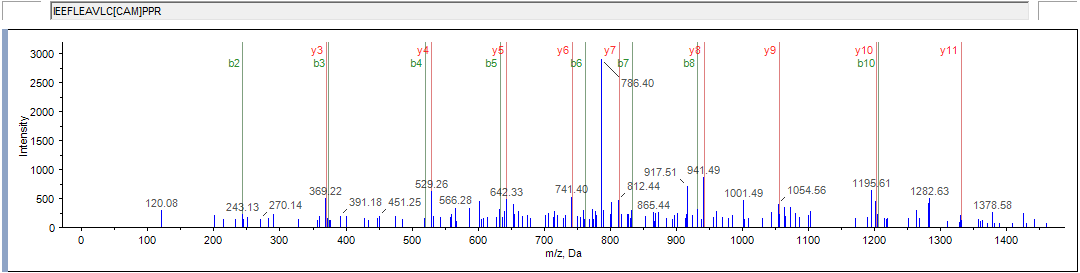


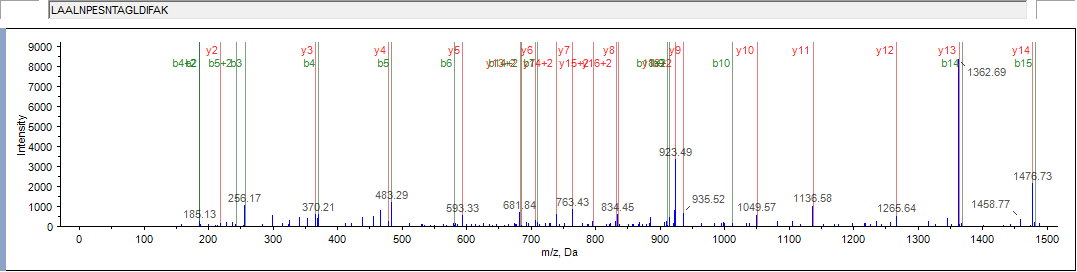


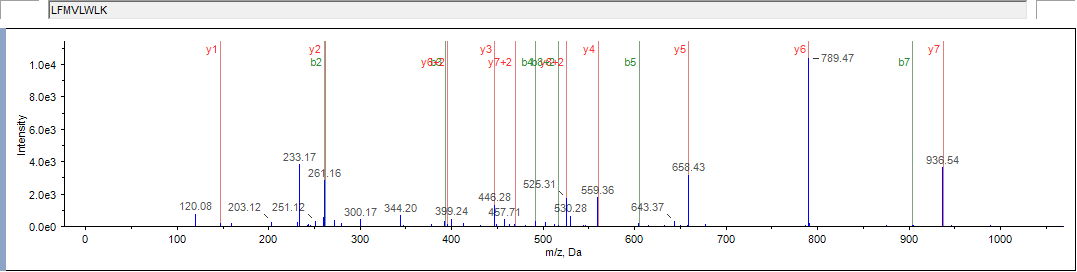


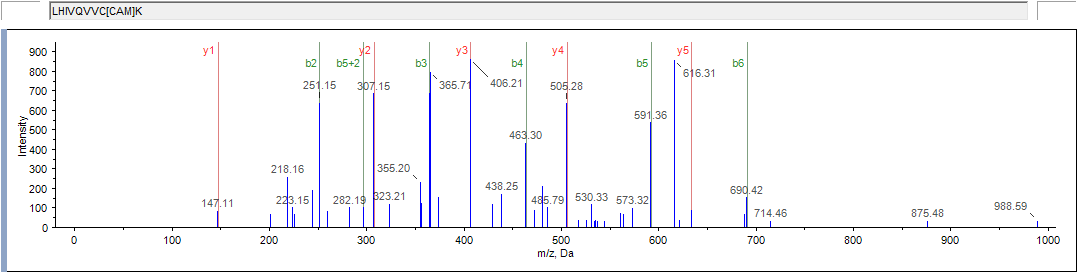


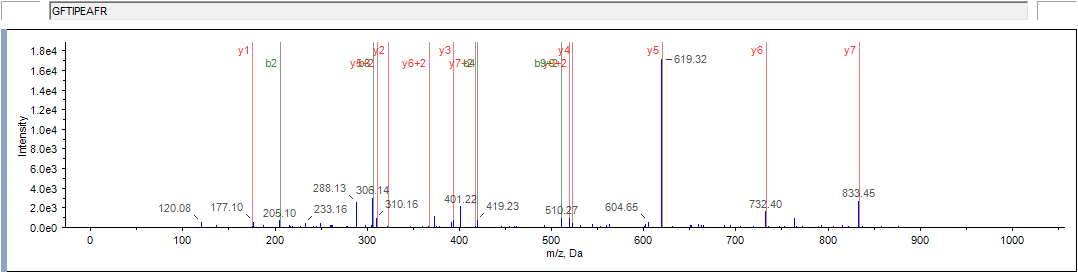


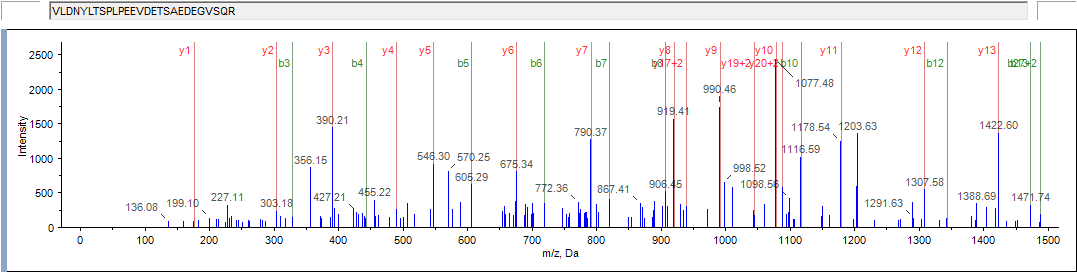


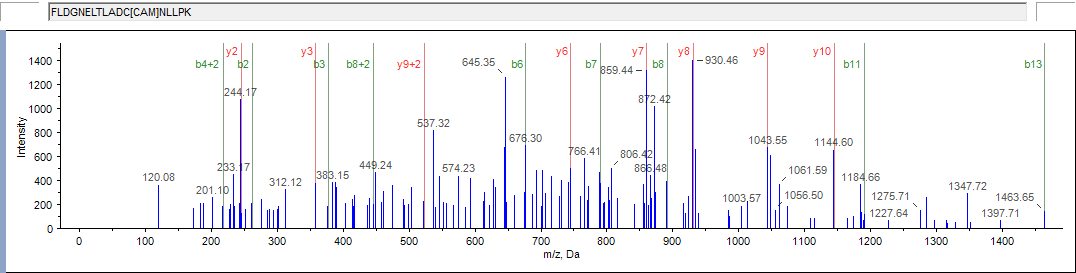


Protein S100-A11

3


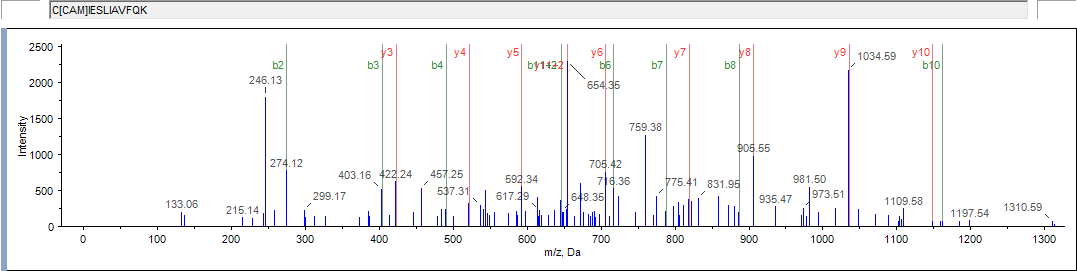


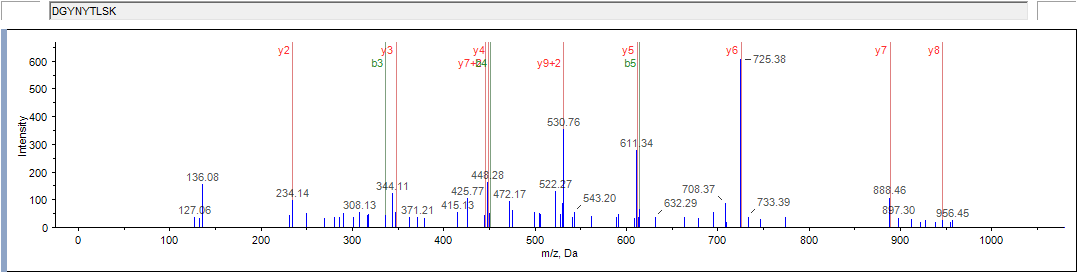


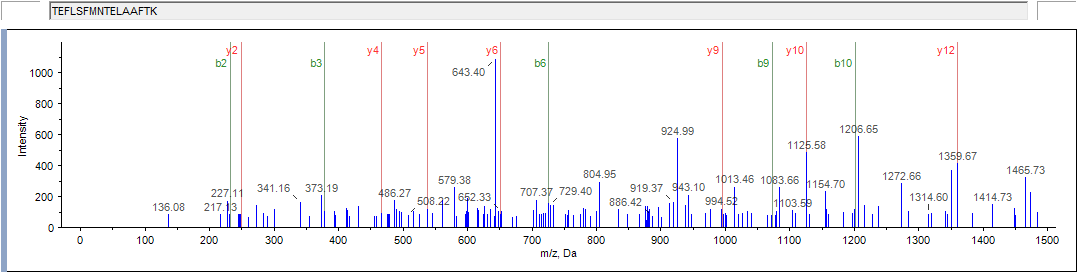


Alpha-enolase

22


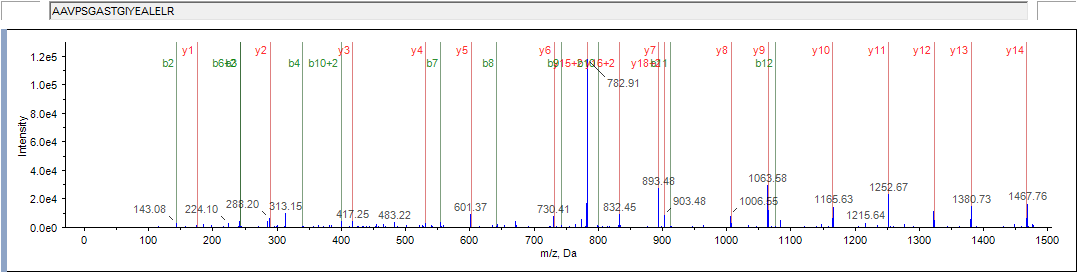


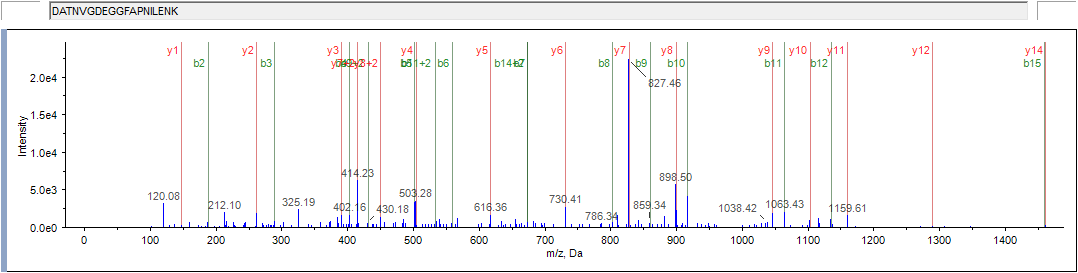


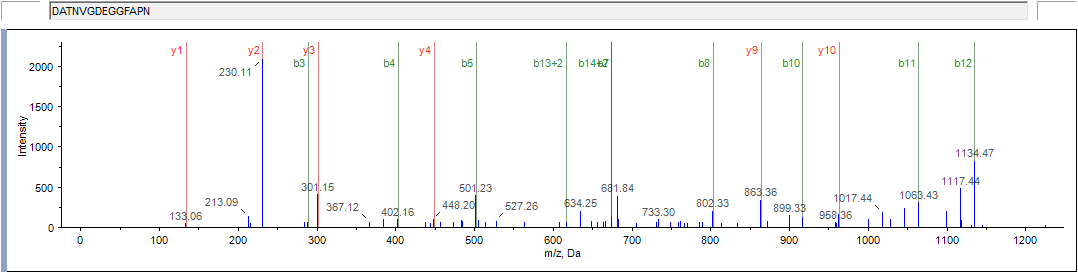


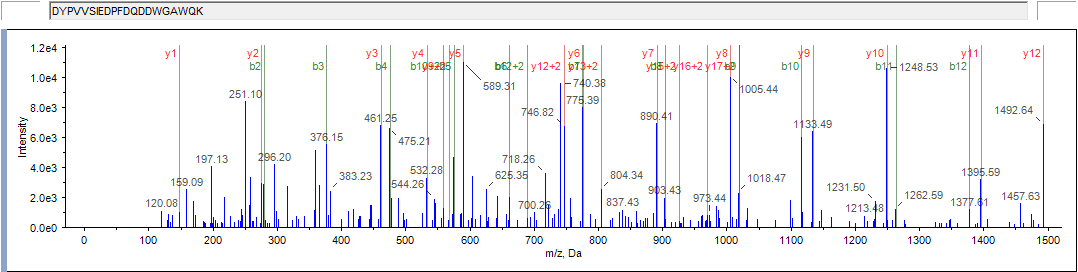


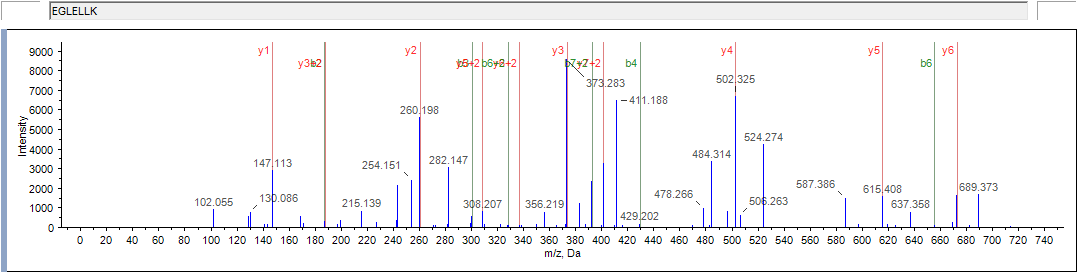


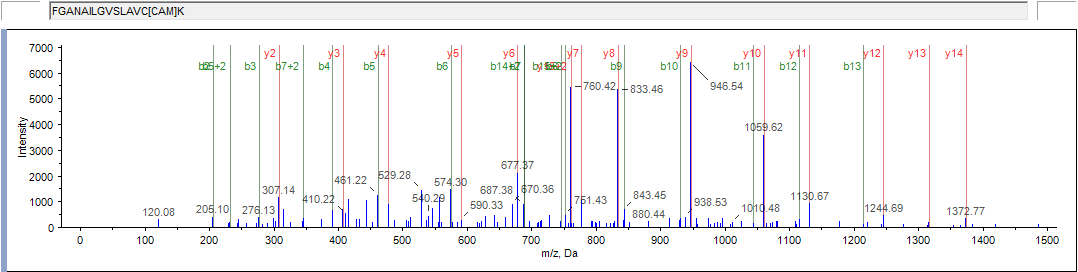


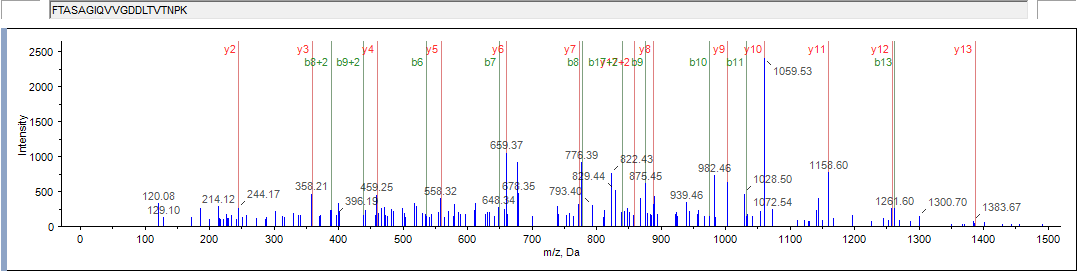


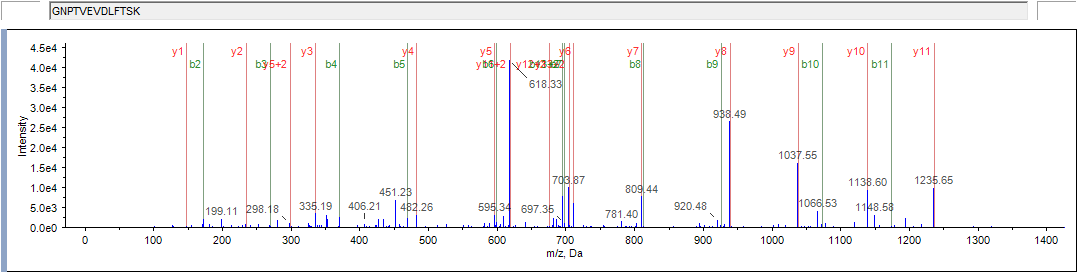


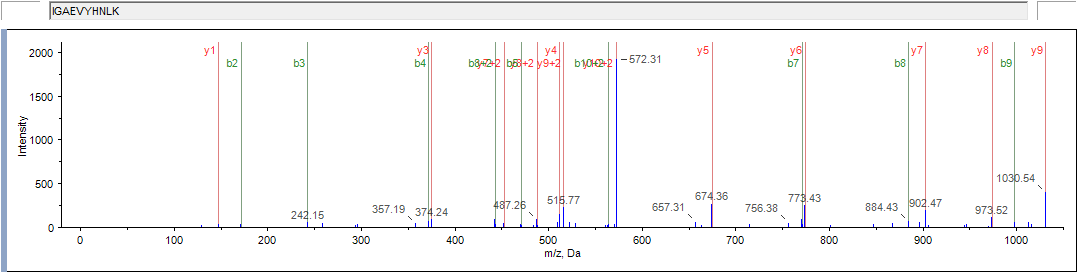


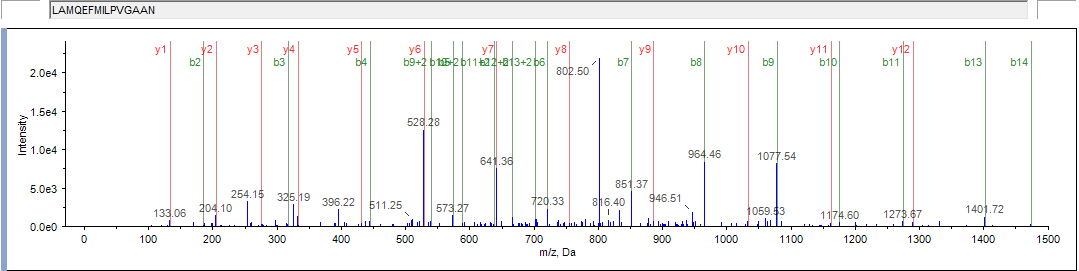


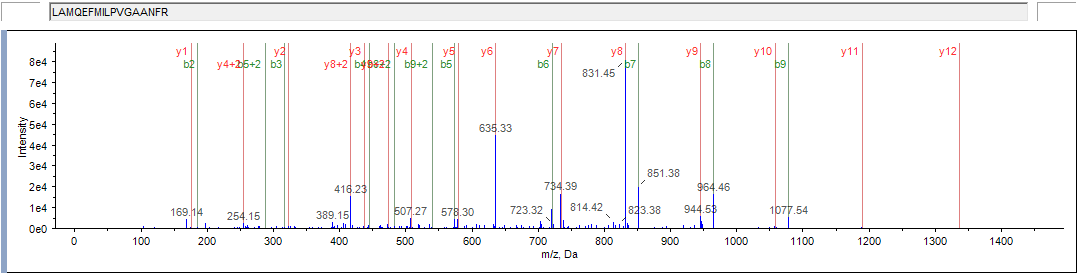


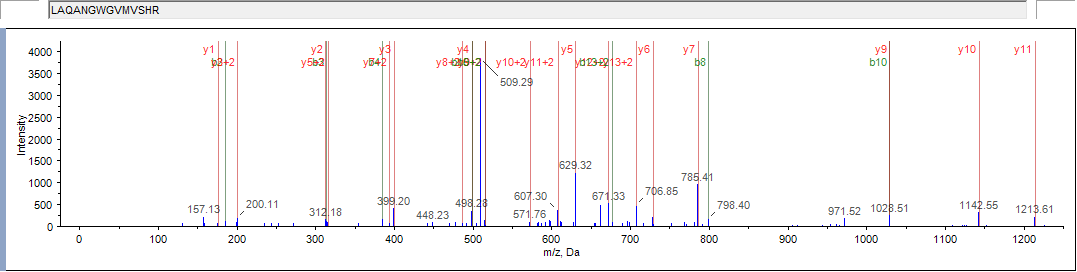


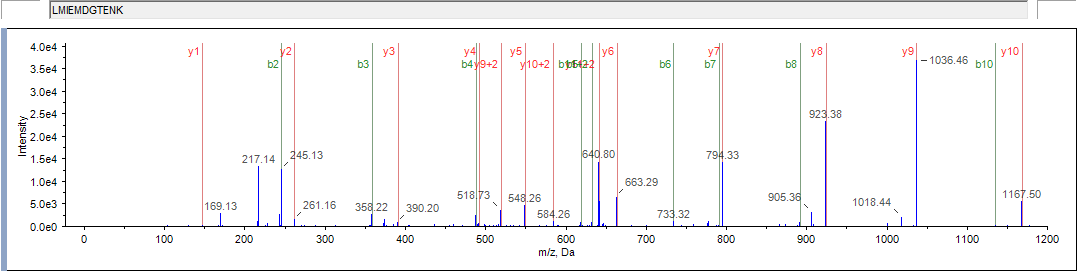


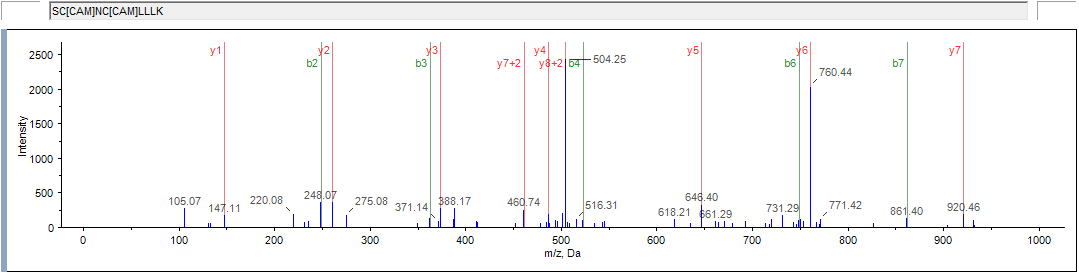


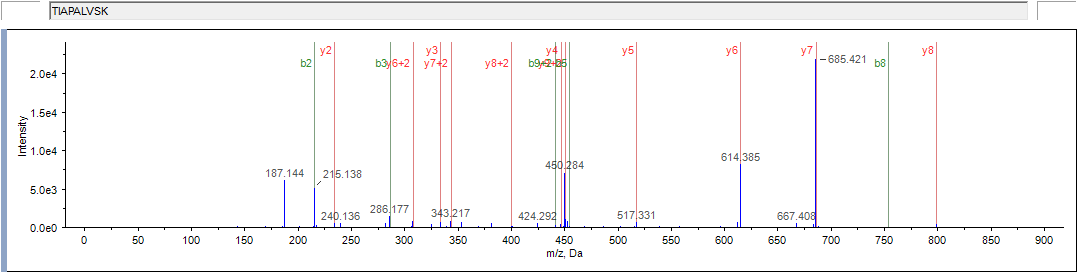


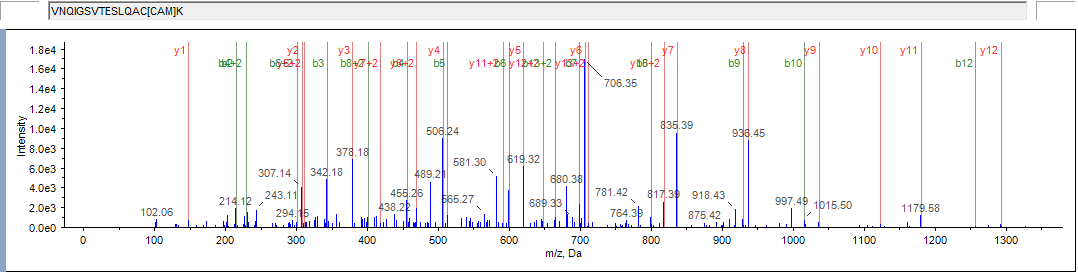


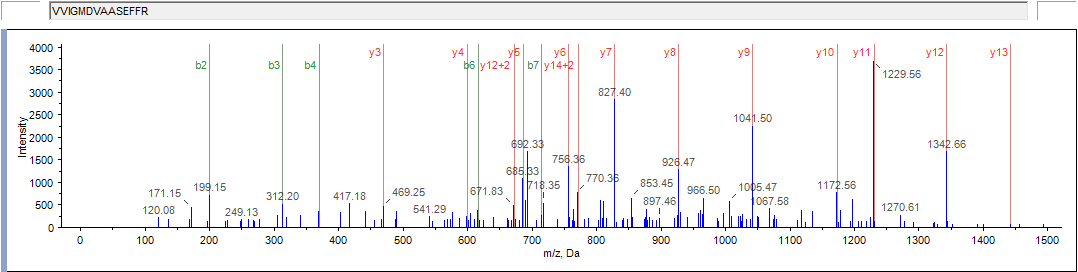


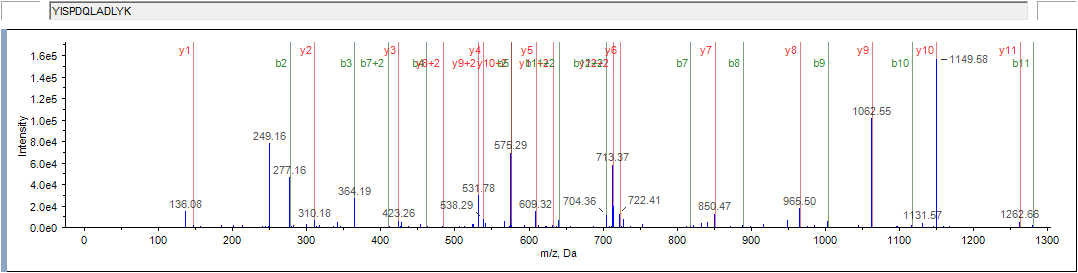


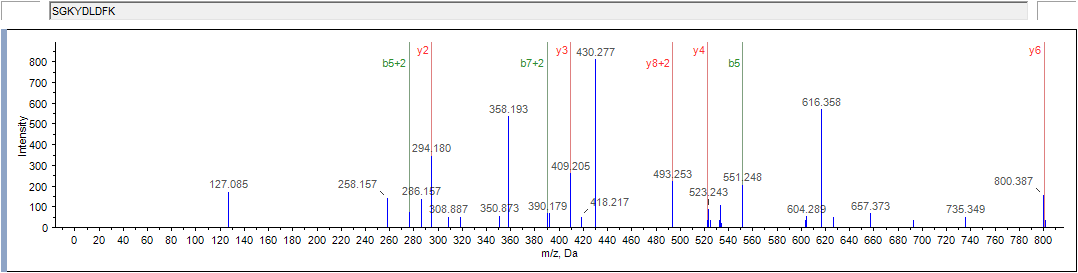


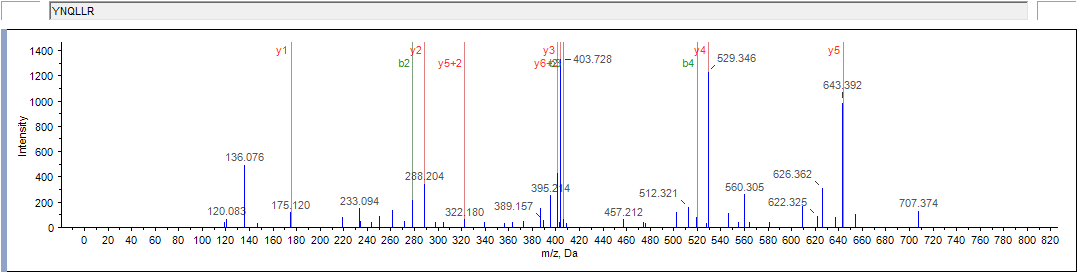


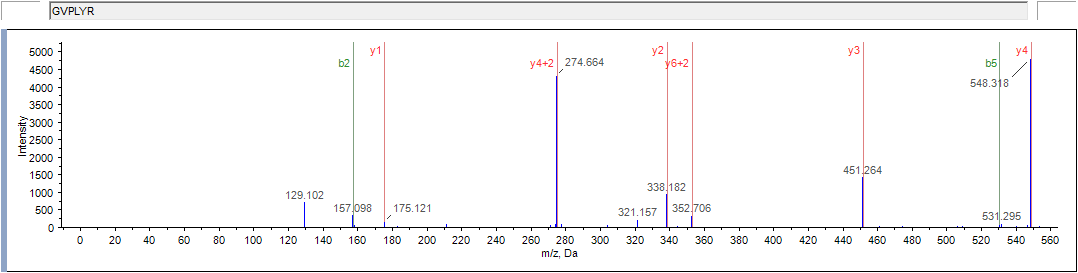


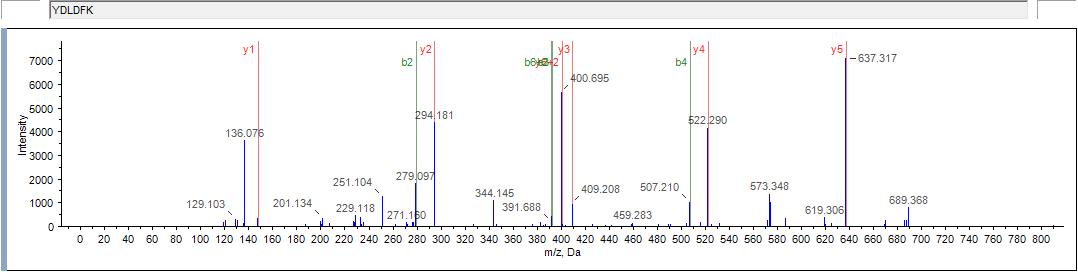


Ubiquitin-conjugating enzyme E2 D2

4


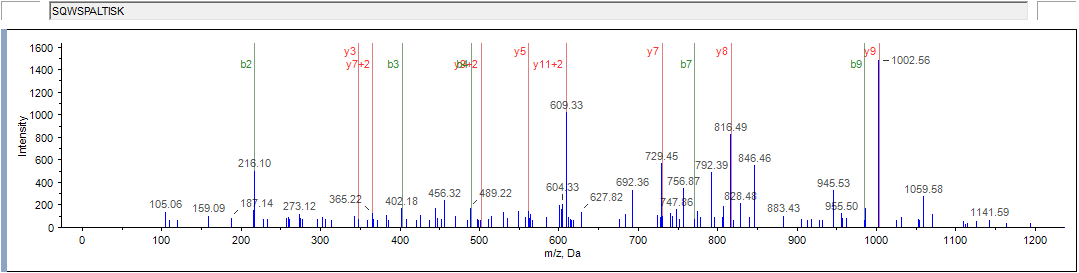


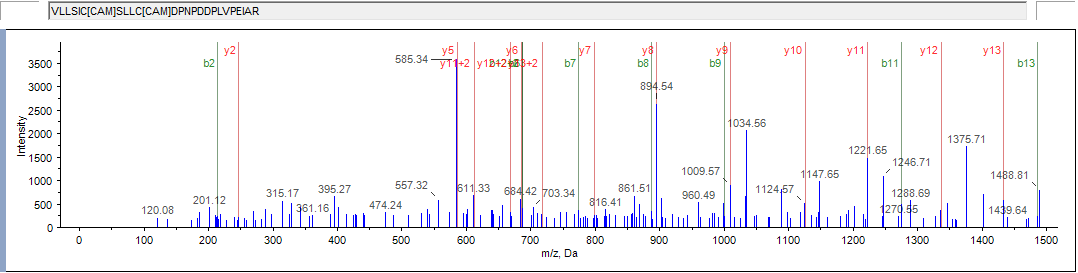


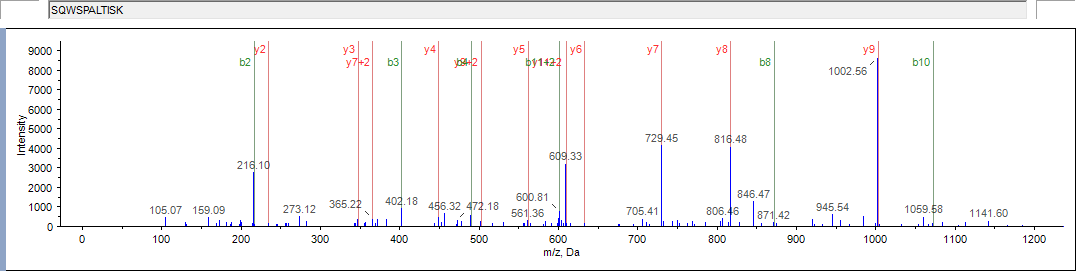


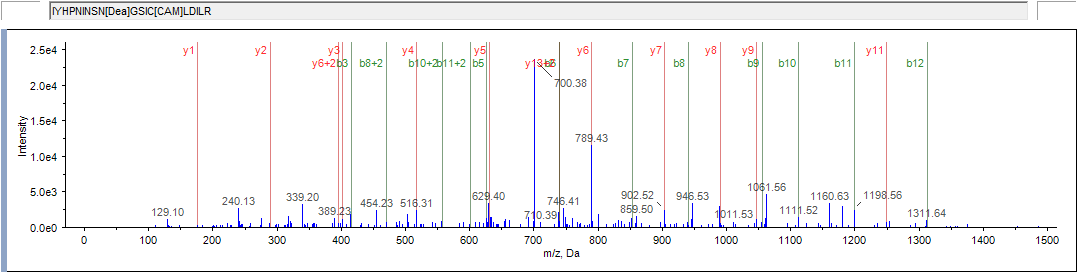


Keratin, type II cytoskeletal 8

9


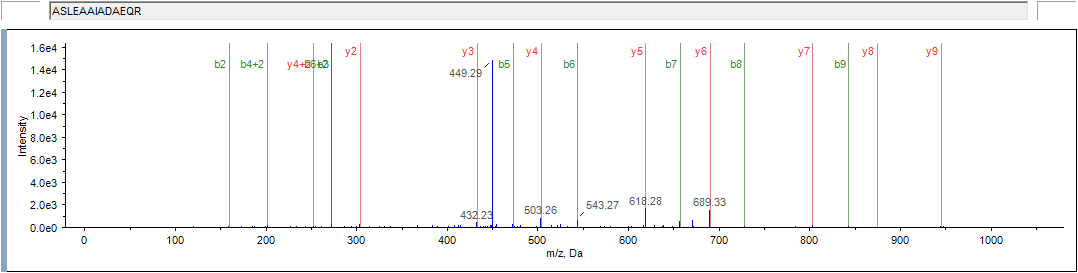


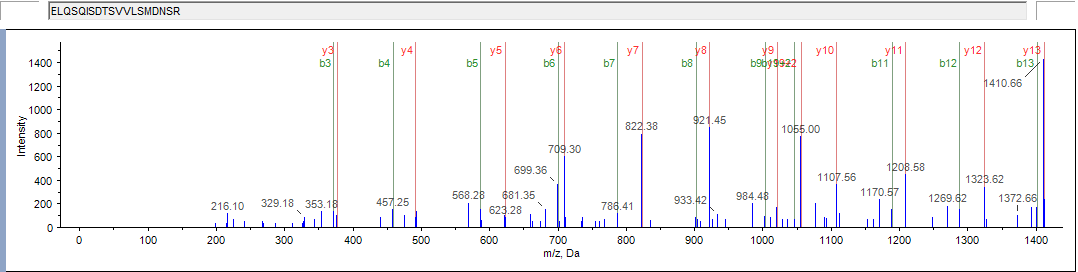


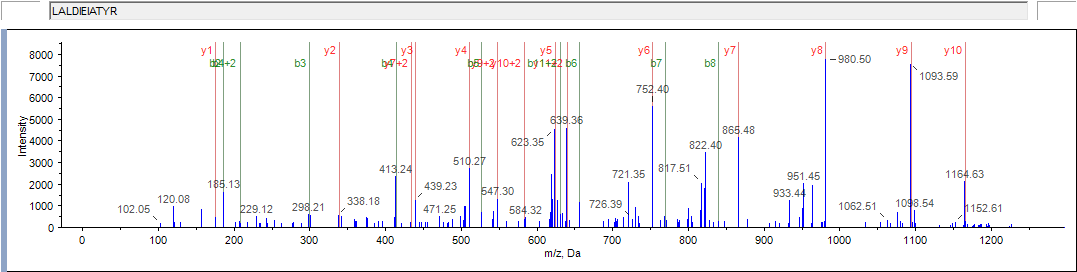


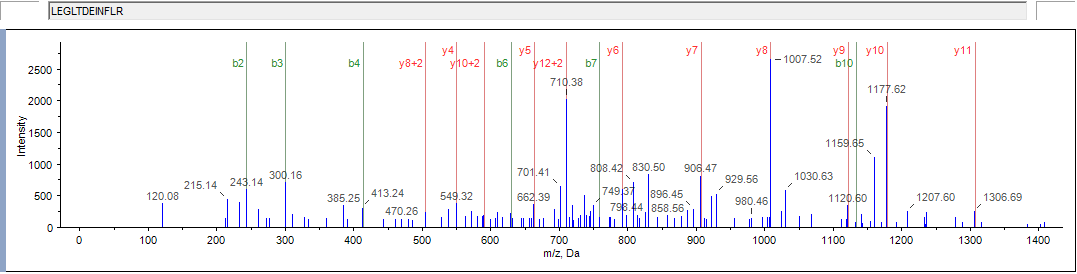


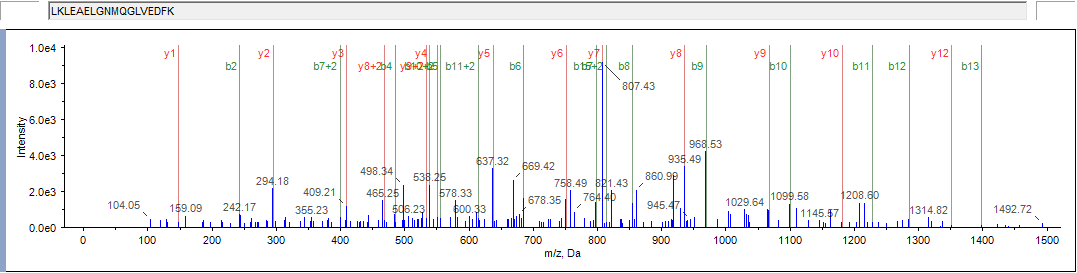


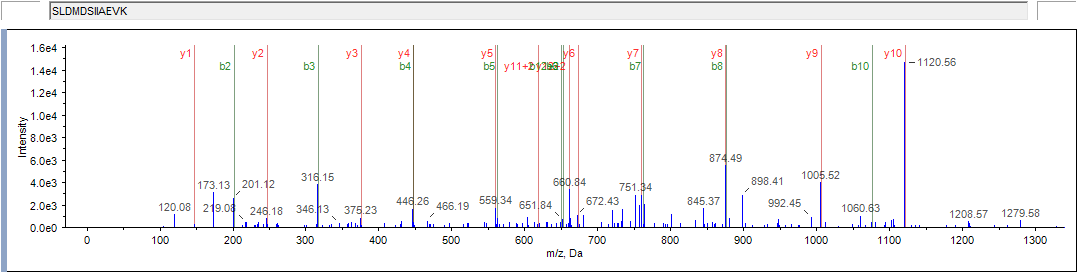


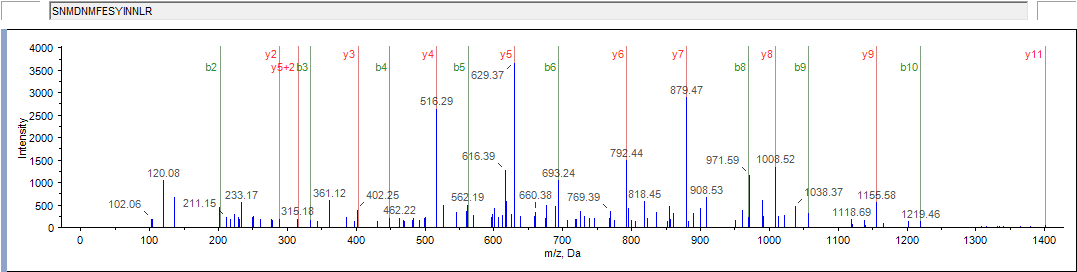


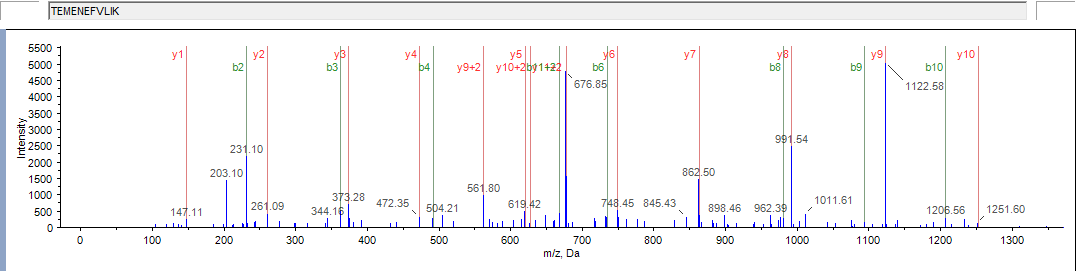


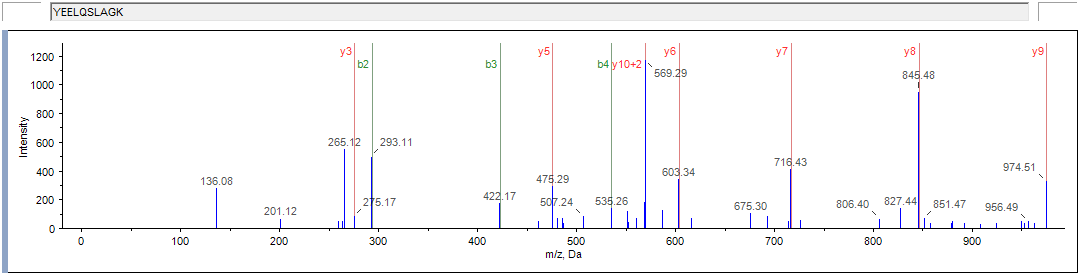


Protein FAM50A

6


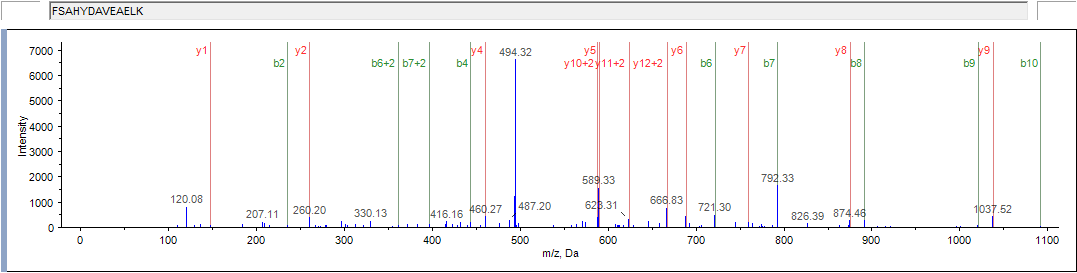


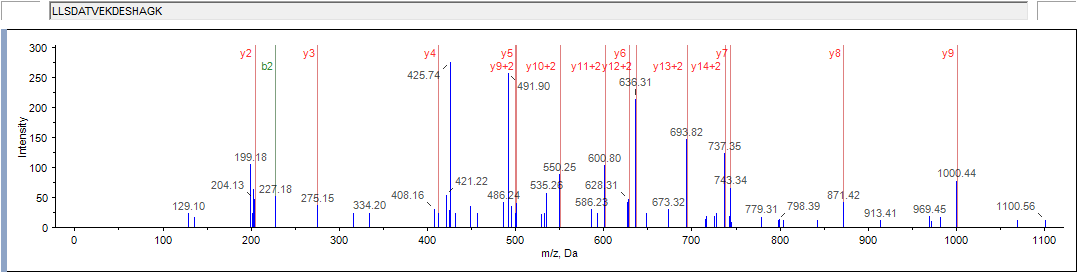


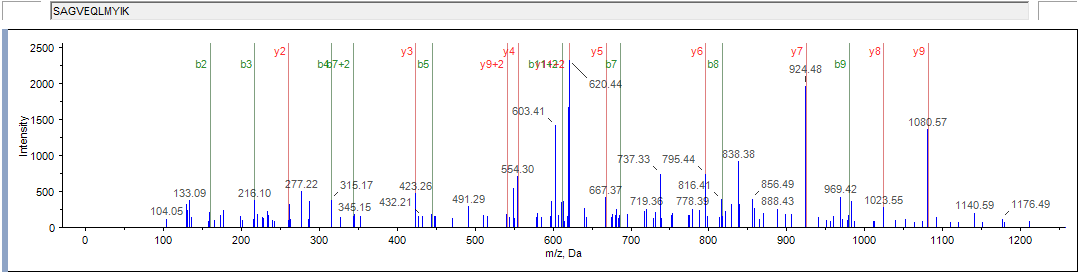


Peptidyl-prolyl cis-trans isomerase D

3

Calreticulin

4

Keratin, type I cytoskeletal 18

11

Protein disulfide-isomerase A3

7

Proteasome subunit beta type-4

2

AP-2 complex subunit alpha-1

7

Prohibitin

2

Inorganic pyrophosphatase

4

ATP synthase subunit beta, mitochondrial

7

Heterogeneous nuclear ribonucleoprotein F

3

Transaldolase

4

Annexin A4

2

Glucose-6-phosphate isomerase

3
